# Risk factors, outcomes, and early prediction of cardiac surgery-associated acute kidney injury: a *post hoc* subgroup analysis of the Epidemiology of Surgery Associated Acute Kidney Injury study^☆^

**DOI:** 10.1016/j.bja.2025.08.043

**Published:** 2025-10-09

**Authors:** Christian Strauß, Felix Albert, Eike Bormann, Daniel T. Engelman, Rinaldo Bellomo, Alexander Zarbock, for the EPIS-AKI investigators

**Affiliations:** 1 Department of Anesthesiology, Intensive Care and Pain Medicine, University Hospital Münster, Muenster, Germany; 2 Institute of Biostatistics and Clinical Research, University of Münster, Muenster, Germany; 3 Heart & Vascular Program, Baystate Health, University of Massachusetts Chan Medical School-Baystate, Springfield, MA, USA; 4 Department of Intensive Care, Austin Hospital, Melbourne, Australia; 5 Australian and New Zealand Intensive Care Research Centre, Monash University, Melbourne, VIC, Australia

**Keywords:** acute kidney injury, cardiac surgery, cardiac surgery-associated acute kidney injury, persistent acute kidney injury, renal failure

## Abstract

**Background:**

Cardiac surgery-associated acute kidney injury (CSA-AKI) is a common and important complication. The risk factors for CSA-AKI remain poorly described. We aimed to identify risk factors for CSA-AKI and develop a risk score for persistent CSA-AKI.

**Methods:**

We performed a *post hoc* subgroup analysis restricted to patients who underwent cardiac surgery within the Epidemiology of Surgery Associated Acute Kidney Injury (EPIS-AKI) study. CSA-AKI was defined as AKI (according to the Kidney Disease: Improving Global Outcomes criteria) within 72 h after surgery. Persistent CSA-AKI was defined as CSA-AKI lasting >48 h. We performed multivariable logistic regression analyses to identify risk factors for CSA-AKI and related outcomes.

**Results:**

The original EPIS-AKI study included 3101 cardiac surgery patients. Of these, 802 (25.9%) developed CSA-AKI. On follow-up, 279 of the 802 patients (34.8%) developed persistent CSA-AKI. We identified independent risk factors for CSA-AKI, moderate/severe CSA-AKI, and persistent CSA-AKI. Patients with persistent CSA-AKI had a higher ICU and hospital mortality compared with patients with transient CSA-AKI. We developed a risk score for predicting persistent CSA-AKI with an area under the receiver operating characteristic curve of 0.79 (95% confidence interval, 0.7355–0.8457).

**Conclusions:**

Overall, 25% of cardiac surgery patients developed cardiac surgery-associated acute kidney injury, and 33% of these patients experienced persistent cardiac surgery-associated acute kidney injury, which was associated with poor outcomes. We developed a risk score for predicting persistent cardiac surgery-associated acute kidney injury, the ‘EPIS CSA-AKI risk score’. Pending further external validation, the score might be used to identify patients who have a high risk for developing persistent cardiac surgery-associated acute kidney injury.

**Editor’s key points**

• In this post hoc subgroup analysis restricted to patients who had cardiac surgery of a large observational study, the authors aimed to identify risk factors for cardiac surgery-associated acute kidney injury (CSA-AKI)
• One-quarter of cardiac surgery patients developed CSA-AKI, which was persistent in about one-third of these patients.
• The authors identified risk factors for CSA-AKI and persistent CSA-AKI and developed a risk score for predicting persistent CSA-AKI. Pending further external validation this risk score might be used to identify patients who have a high risk for developing persistent CSA AKI.

Cardiac surgery-associated acute kidney injury (CSA-AKI) is a common and clinically important complication.^1^^,^^2^ It is associated with worse short- and long-term outcomes,^2^, ^3^, ^4^ including increased hospital mortality,^5^ prolonged length stay in the hospital,^6^ the development of chronic kidney disease,^7^ and accelerated progression to end-stage renal disease.^8^ Recent studies demonstrated that both the duration and severity of CSA-AKI have important adverse implications for kidney recovery and patient outcomes.^9^ Episodes of persistent CSA-AKI seem to have a particularly strong association with adverse patient trajectories.^9^^,^^10^

Numerous interventions to reduce CSA-AKI have been investigated; however, despite recent breakthroughs,^11^ few approaches have proven efficacious.^12^ The identification of modifiable and non-modifiable risk factors and risk scores for CSA-AKI may be helpful to identify patients at risk and to target interventions. Previous studies have identified several important risk factors for CSA-AKI.^7^^,^^12^^,^^13^ However, these studies, and previous efforts in creating a prognostic score,^14^ were based on retrospective data and had limited data granularity. Consequently, we performed a *post hoc* subgroup analysis restricted to patients who underwent cardiac surgery within the Epidemiology of Surgery Associated Acute Kidney Injury (EPIS-AKI) study to identify risk factors for CSA-AKI and develop a risk score for persistent CSA-AKI.

## Methods

### Data collection

All analysed data were derived from the EPIS-AKI study dataset. The recently published EPIS-AKI study was an international multicentre prospective study to investigate the incidence of postoperative AKI after major surgery.^15^ It included 10 000 patients from 30 countries. The original study only included patients who had major surgery lasting more than 2 h and who were treated in an ICU after surgery. This *post hoc* subgroup analysis of the EPIS-AKI study was restricted to patients who underwent cardiac surgery.

### Outcomes

In this substudy, we investigated outcomes in patients with CSA-AKI, identified risk factors for CSA-AKI, and developed a risk score to predict persistent CSA-AKI.

CSA-AKI was defined as AKI (according to the Kidney Disease: Improving Global Outcomes [KDIGO] criteria) within 72 h after surgery.^16^ Transient CSA-AKI was defined as CSA-AKI lasting ≤48 h, and persistent CSA-AKI was defined as CSA-AKI lasting >48 h.^17^

Additional outcomes were use of renal replacement therapy (RRT) in the ICU, use of RRT during the hospital stay, length of stay in the ICU and in the hospital, and ICU and in-hospital mortality. All longer-term outcomes were censored at day 90. In addition, the variables on length of stay were only analysed in patients who survived for at least 90 days to avoid biased results owing to different mortality distributions between groups.

On the basis of their predictive weight, we selected the six factors that were most strongly associated with the development of persistent CSA-AKI and created a prediction score. Systemic inflammatory response syndrome (SIRS) was defined by the occurrence of at least two of the following conditions: (1) body temperature <36°C or >38°C, (2) heart rate >90 beats min^−1^, (3) tachypnea with a respiratory rate >20 bpm and a paCO_2_ ≤4.4 kPa or an oxygenation index <200 (under mechanical ventilation), or (4) leucocyte count <4000 mm^−3^, >12 000 mm^−3^, or >10% immature leucocytes. Haemodynamic instability was defined as ‘need for vasopressors beyond surgery’. ‘Postoperative bleeding’ was defined by a postoperative decrease in haemoglobin that triggered the transfusion of red blood cells.

### Statistical analysis

Frequencies, percentages, medians, quartiles, and *P*-values were calculated for the baseline variables and outcomes as applicable. Fisher's exact test and Pearson’s χ^2^ test were used to compare categorical variables between groups. Continuous variables were compared using the Mann–Whitney *U*-test. Confidence intervals (CIs) for binomial proportion estimates were calculated using the Clopper–Pearson exact method with a 95% confidence level. Multivariable logistic regression analyses were performed to identify and assess the association of risk factors for CSA-AKI.

Missing data were imputed using k-nearest neighbour imputation. For each outcome, we then excluded categorical variables with categories in which the outcome did not occur and multicollinear variables from further analysis. We included all the remaining variables in a logistic regression model and then performed a fast backward variable selection based on Akaike’s information criterion to identify a reasonable set of potential risk factors for the outcome. In each iteration, the influencing variable whose exclusion caused the greatest reduction of the Akaike’s information criterion compared with the current model was excluded from the current model until no omission of a single variable resulted in a further reduction of the Akaike’s information criterion. The reduced models are reported with odds ratios, corresponding 95% CIs, and *P*-values.

For the risk score for predicting persistent CSA-AKI, the dataset was first split into a training set (70%) and a validation set (30%) stratified by persistent CSA-AKI. Variable selection was performed using a stepwise selection based on the significance level on the training set. The selected variables were used in a generalised mixed linear regression model with logit as link function and centre as a random effect to create an easy to use and interpret risk score for the occurrence of persistent CSA-AKI. The score was created as described by Sullivan and colleagues.^18^ An area under the receiver operating characteristic (AUROC) curve and calibration curve for the score on the validation set were estimated. All *P*-values and confidence limits were two sided and not adjusted for multiple testing. *P*-values are therefore regarded statistically noticeable (‘significant’) in case *P*≤0.05. An overall significance level across all statistical analyses was not determined and cannot be calculated. Analyses were conducted using R (version R-4.3.1; R Foundation for Statistical Computing, Vienna, Austria) and SAS 9.4 (SAS Institute, Cary, NC, USA).

## Results

### Patient characteristics

This substudy included 3101 cardiac surgery patients. Patient characteristics and surgical data are shown in Table 1 and Supplementary Table 1.

**Table 1:** Patient and baseline characteristics. Data are median (Q1, Q3), *n* (%), or *n*/*N* (%).ACEi/ARB, angiotensin-converting-enzyme inhibitor/aldosterone receptor blocker; CKD, chronic kidney disease; COPD, chronic obstructive pulmonary disease; CSA-AKI, cardiac surgery-associated acute kidney injury; GFR, glomerular filtration rate; IDDM, insulin-dependent diabetes mellitus; NIDDM, non-insulin-dependent diabetes mellitus; NYHA, New York heart association, NSAIDs, non-steroidal ant-inflammatory drugs.

	All patients	No CSA-AKI	CSA-AKI	Significance
	(*n*=3101)	(*n*=2299)	(*n*=802)	
Baseline characteristics				
Age (yr)	65 (57, 71)	63 (56, 70)	68 (60, 74)	<0.001
Male	2195 (70.8)	1619 (70.4)	576 (71.8)	0.469
Height (cm)	170 (163, 176) (missing 3)	170 (163, 176) (missing 3)	170 (163, 176)	0.880
Weight (kg)	80 (70, 90) (missing 2)	79 (70, 90) (missing 2)	80 (70, 91)	0.006
BMI	27.5 (24.8, 30.8) (Missing 3)	27.4 (24.6, 30.6) (Missing 3)	27.7 (25, 31.3)	0.005
Serum creatinine (mg dl^−1^)	0.9 (0.8, 1.1) (missing 4)	0.9 (0.8, 1) (missing 4)	1 (0.8, 1.2)	<0.001
United Nations geoscheme				<0.001
Africa	135/3101 (4.4)	115/2299	20/802 (2.5)	
Asia	918/3101 (29.6)	715/2299 (31.1)	203/802 (25.3)	
Europe	1800/3101 (58)	1317/2299 (57.3)	483/802 (60.2)	
North America	186/3101 (6)	98/2299	88/802 (11)	
South America	62/3101 (2)	54/2299 (2.3)	8/802 (1)	
Health expenditure group				<0.001
Low	934/3088 (30.2)	747/2292 (32.6)	187/796 (23.5)	
Medium	1211/3088 (39.2)	985/2292 (43)	226/796 (28.4)	
High	943/3088 (30.5)	560/2292 (24.4)	383/796 (48.1)	
Comorbidities				
Hypertension	2294/3101 (74)	1648/2292 (71.7)	646/802 (80.5)	<0.001
Diabetes mellitus				
Total	922/3101 (29.7)	638/2299 (27.8)	284/802 (35.4)	<0.001
Diabetes type				0.121
IDDM	201/922 (21.8)	130/638 (20.4)	71/284 (25)	
NIDDM	721/922 (78.2)	508/638 (79.6)	213/284 (75)	
Congestive heart failure	1407/3101 (45.4)	996/2299 (43.3)	411/802 (51.2)	<0.001
NYHA stage				<0.001
NYHA I	381/1407 (27.1)	335/996 (33.6)	46/411 (11.2)	
NYHA II	486/1407 (34.5)	351/996 (35.2)	135/411 (32.8)	
NYHA III	493/1407 (35)	284/996 (28.5)	209/411 (50.9)	
NYHA IV	47/1407 (3.3)	26/996 (2.6)	21/411 (5.1)	
Previous myocardial infarction	876/3101 (28.2)	645/2299 (28.1)	231/802 (15.2)	0.682
Peripheral vascular disease	424/3101 (13.7)	302/2299 (13.5)	122/802 (15.2)	0.152
Atrial flutter/fibrillation	498/3101 (16.1)	310/2299 (13.5)	188/802 (23.4)	<0.001
COPD	275/3101 (8.9)	168/2299 (7.3)	107/802 (13.3)	<0.001
CKD (GFR <60 ml min^−1^)	332/3101 (10.7)	180/2299 (7.8)	152/802 (19)	<0.001
Previous stroke	206/3101 (6.6)	139/2299 (6)	67/802 (8.4)	0.026
ASA physical status				<0.001
1	32/3101 (1)	26/2299 (1.1)	6/802 (0.7)	
2	834/3101 (26.9)	717/2299 (31.2)	117/802 (14.6)	
3	1687/3101 (54.4)	1227/2299 (53.4)	460/802 (57.4)	
4	548/3101 (17.7)	329/2299 (14.3)	219/802 (27.3)	
Medication				
ACEi or ARBs	1770/3101 (57.1)	1274/2299 (55.4)	496/802 (61.8)	0.002
Beta-blockers	1905/3101 (61.4)	1420 (61.8)	485/802 (60.5)	0.528
Aspirin	1830/3101 (59)	1357/2299 (59)	473/802 (59)	1.000
Statins	1704/3101 (55)	1240/2299 (53.9)	464/802 (57.9)	0.058
Diuretics	1052/3101 (33.9)	697/2299 (30.3)	355/802 (44.3)	<0.001
Use of contrast media (1 week before surgery)	835/3101 (26.9)	612/2299 (26.6)	223/802 (27.8)	0.518
NSAIDs	121/3101 (3.9)	88/2299 (3.8)	33/802 (4.1)	0.751
Vasopressors	34/3101 (1.1)	22/2299 (1)	12/802 (1.5)	0.236

### Cardiac surgery-associated acute kidney injury

Of the 3101 included cardiac surgery patients, 802 (25.9%) developed CSA-AKI (Table 2). Of these 802 patients with CSA-AKI, 487 (60.7%) developed KDIGO stage 1, 211 (26.3%) KDIGO stage 2, and 104 (13%) KDIGO stage 3 CSA-AKI. Of the 802 patients with CSA-AKI, 389 patients (48.5%) were diagnosed with CSA-AKI based on an elevation of serum creatinine (SCr), 225 patients (28%) based on reduced urinary output (UO), and 188 patients (23.4%) based on both. As Supplementary Table 2 shows, AKI was transient in 523 patients and persisted in 279 patients.

**Table 2:** CSA-AKI severity and other outcomes. Data are *n*/*N* (%) or median (Q1, Q3) . CRRT, continuous renal replacement therapy; CSA-AKI, cardiac surgery-associated acute kidney injury; D90, day 90; IHD, intermittent haemodialysis; KDIGO, Kidney Disease Improving Global Outcomes; PIRRT, prolonged renal replacement therapy; RRT, renal replacement therapy.

	All patients	No CSA-AKI	CSA-AKI	Significance
	(*n*=3101)	(*n*=2299)	(*n*=802)	
Cardiac surgery-associated acute kidney injuryb severity				
KDIGO 1				
All			487/802 (60.7)	<0.001
Serum creatinine			307/487 (63.0)	
Urine output			111/487 (22.8)	
Both			69/487 (22.8)	
KDIGO 2				
All			211/802 (26.3)	<0.001
Serum creatinine			57/211 (27.0)	
Urine output			91/211 (43.1)	
Both			63/211 (29.9)	
KDIGO 3				
All			104/802 (13.0)	<0.001
Serum creatinine			25/104 (24.0)	
Urine output			23/104 (22.1)	
Both			56/104 (53.6)	
RRT in ICU/postoperative				
All	101/3101 (3.3)	1/2299 (0.0)	100/802 (12.5)	
RRT modality				
CRRT		1/1 (100.0)	79/100 (79.0)	
IHD		0/1 (0.0)	8/100 (8.0)	
PIRRT		0/1 (0.0)	13/100 (13.0)	
RRT during hospital stay				
All	110/3101 (3.6)	4/2292 (0.2)	106/802 (13.2)	<0.001
KDIGO 1			7/487 (1.4)	
KDIGO 2			35/211 (16.6)	
KDIGO 3			64/104 (61.5)	
>72 h		4/4 (100.0)		
Mortality				
D90 general mortality	88/3101 (2.8)	25/2299 (1.1)	63/802 (7.9)	<0.001
CSA-AKI severity				
KDIGO 1			16/487 (3.3)	<0.001
KDIGO 2			17/211 (8.1)	
KDIGO 3			30/104 (28.8)	
ICU mortality	46/3101 (1.5)	10/2299 (0.4)	36/802 (4.5)	
CSA-AKI severity				
KDIGO 1			4/487 (0.8)	<0.001
KDIGO 2			10/211 (4.7)	
KDIGO 3			22/104 (21.2)	
Hospital mortality	67/3101 (2.2)	17/2299 (0.7)	50/802 (6.2)	
CSA-AKI severity				
KDIGO 1			8/487 (1.6)	
KDIGO 2			12/211 (5.7)	
KDIGO 3			30/104 (28.8)	
Length of stay, in D90 survivors (days)				
ICU		2 (1, 3)	3 (2, 5)	<0.001
CSA-AKI severity				
KDIGO 1			3 (1, 4)	<0.001
KDIGO 2			4 (2, 7)	
KDIGO 3			5 (2.8, 12.2)	
Hospital		12 (9, 16)	13 (9, 20)	
CSA-AKI severity				
KDIGO 1			12 (9, 18)	<0.001
KDIGO 2			13 (8, 21.8)	
KDIGO 3			18.5 (12.2, 30.0)	

### Secondary outcomes

**Fig 1:**
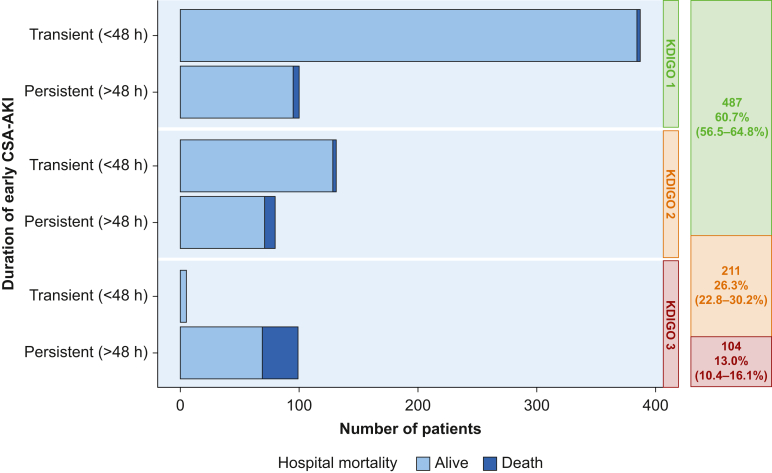
Statistical representation of the in-hospital mortality of CSA-AKI patients separated by the highest reached stages within 72 h postoperative, and AKI duration. The X-axis on the right side represents the number of patients and percentage in each AKI stage, identified by colour (green as stage 1, orange as stage 2 and red as stage 30). CSA-AKI, cardiac surgery associated acute kidney injury; KDIGO, Kidney Disease Improving Global Outcomes.

Of the 3101 patients, 110 (3.6%) were treated with RRT during hospital stay (Table 2). In patients with CSA-AKI, the RRT rate was 12.5% during the ICU stay and 13.2% during the hospital stay. The following outcomes are shown in Figure 1 and Table 2: length of stay in the ICU and in the hospital, ICU mortality, in-hospital mortality, and 90-day mortality. Compared with patients without CSA-AKI, patients with CSA-AKI stayed longer in the ICU (2 [1, 3] days *vs* 3 [2, 5] days) and in the hospital (12 [9, 16] days *vs* 13 [9, 20] days) and had higher ICU (0.4% *vs* 4.5%), in-hospital (0.7% *vs* 6.2%), and 90-day (1.1% *vs* 7.9%) mortality. AKI was significantly associated with longer length of stay in the ICU and hospital, ICU mortality, in-hospital mortality, and 90-day mortality when adjusted for other factors (Supplementary Tables 6–11).

Ninety-day mortality increased with increasing severity of CSA-AKI (Table 2). The relationship between AKI severity and mortality was stronger in patients with persistent CSA-AKI (Fig. 1). As shown in Figure 1 and additional Supplementary Figures 1–3, the occurrence of a persistent CSA-AKI increased the probability of adverse outcomes for all analysed endpoints, including higher RRT rate and hospital mortality.

### Risk factors for cardiac surgery-associated acute kidney injury

Multivariable regression analysis revealed independent associations between CSA-AKI and non-modifiable risk factors, including age, baseline SCr, pre-existing comorbidities (diabetes mellitus and atrial fibrillation), cardiopulmonary bypass (CPB) time, intraoperative use of vasopressors, intraoperative bleeding, and postoperative complications (haemodynamic instability, bleeding, re-operation, and pneumonia) (Supplementary Table 3). Modifiable risk factors were use of the cell saver, intraoperative use of aminoglycosides, intraoperative use of diuretics, postoperative fluid balance until day 3, and postoperative use of nephrotoxic drugs and diuretics.

### Risk factors for moderate/severe cardiac surgery-associated acute kidney injury

Development of severe AKI (KDIGO stage 2 and 3) was independently associated with non-modifiable risk factors, including age, baseline BMI, baseline SCr, diabetes mellitus, chronic obstructive pulmonary disease, intraoperative bleeding complications, intraoperative pulmonary complications, and postoperative complications (haemodynamic instability, re-operation, and SIRS) (Supplementary Table 4). Potentially modifiable risk factors were intraoperative use of the cell saver, intraoperative application of aminoglycosides and vancomycin, and increasing postoperative fluid balance until day 3.

### Risk factors for persistent cardiac surgery-associated acute kidney injury

Development of persistent CSA-AKI was independently associated with non-modifiable risk factors, including age, BMI, baseline SCr, hypertension, chronic kidney disease at baseline, CPB time, and postoperative complications (haemodynamic instability, bleeding, re-operation, and SIRS) (Supplementary Table 5). Intraoperative use of aminoglycosides, intraoperative use of diuretics, postoperative fluid balance until day 3, and postoperative use of diuretics were identified as modifiable risk factors.

### Risk prediction and scoring tool

**Fig 2:**
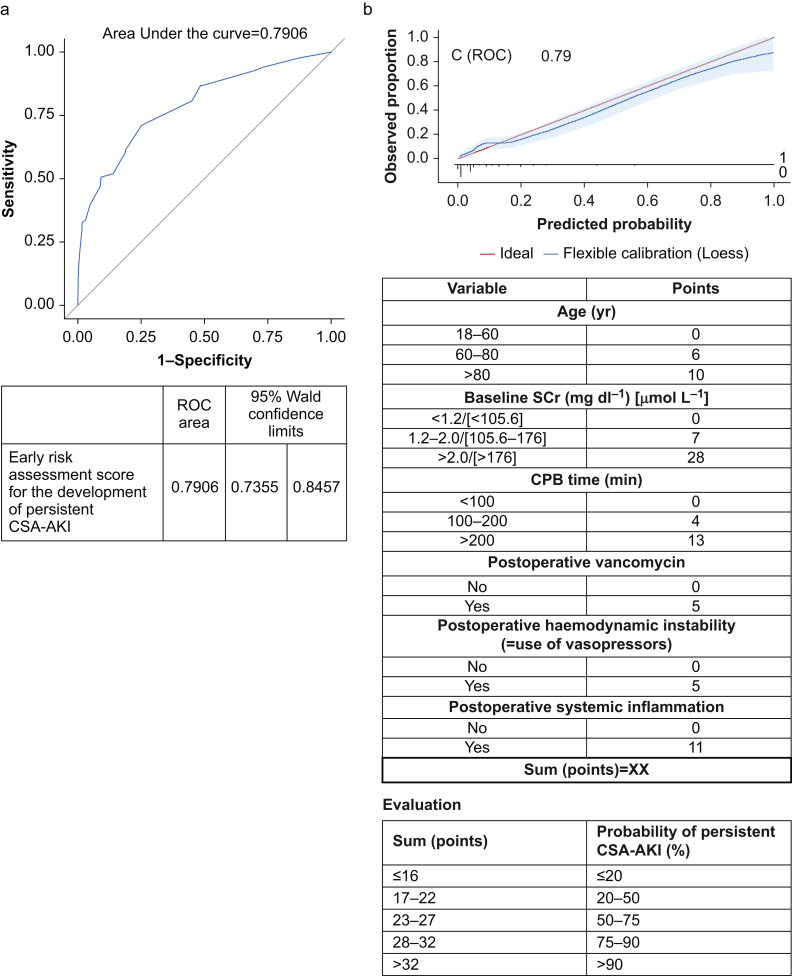
Early risk assessment score for the development of persistent CSA-AKI. (a) ROC curve for early assessment score for the development of persistent AKI. (b) Score. CPB, cardiopulmonary bypass; CSA-AKI, cardiac surgery associated acute kidney injury; ROC, receiver operating characteristic; SCr, serum creatinine.

Some risk factors were identical for all investigated entities of CSA-AKI; other risk factors were specific to one entity. On the basis of these data, we developed a risk score for the prediction of persistent CSA-AKI, consisting of six factors. The score includes age (years), baseline SCr (mg dl^−1^/μmol L^−1^), CPB time (minutes), use of postoperative vancomycin, postoperative haemodynamic instability (defined by a need for vasopressors beyond surgery), and postoperative SIRS. The AUROC on the validation set was 0.79 (95% CI, 0.7355–0.8457) for predicting persistent CSA-AKI within 72 h after cardiac surgery (Fig. 2).

## Discussion

In this *post hoc* substudy of a prospective observational study, we found that ∼25% of all patients undergoing cardiac surgery developed CSA-AKI in the first 3 days. In 33% of these patients, CSA-AKI was moderate or severe. Persistent CSA-AKI occurred in 33% of CSA-AKI patients and was associated with worse outcomes. We developed a risk score to predict the development of persistent CSA-AKI, which carried an AUROC of 0.79.

It is known that CSA-AKI is associated with worse outcomes.^13^^,^^19^ Even mild or moderate renal impairment after cardiac surgery is a predictor for increased hospital mortality and decreased 3-yr survival.^5^ The outcome worsens if patients require RRT.^20^ In line with the published literature,^21^ we confirmed that the severity and persistence of CSA-AKI were associated with worse outcomes. We identified different modifiable risk factors that are associated with the development of CSA-AKI, and we demonstrate that different risk factors exist for different AKI types.

Up to date, no causal treatments for CSA-AKI have been discovered. Yet several preventive approaches have shown promising results.^11^^,^^22^^,^^23^ Thus, prevention should be considered key to improving outcomes.^13^^,^^24^^,^^25^ CSA-AKI is a syndrome and the occurrence is influenced by susceptibility (e.g. comorbidities) and exposure (e.g. CPB time).^25^ Knowledge about modifiable factors might help reduce the occurrence of CSA-AKI and subsequently improve patients’ outcomes. Optimising risk factors might reduce the occurrence of AKI after cardiac surgery.^26^, ^27^, ^28^ In addition, we identified nephrotoxic drugs as modifiable factors. The use of these drugs should be avoided, if possible, in patients at high risk for CSA-AKI or with an already established CSA-AKI.^16^ In these patients, alternative drugs can be used. Instead of non-steroidal ant-inflammatory drugs for postoperative pain therapy, paracetamol/acetaminophen can be used or a non-nephrotoxic antibiotic can be given instead of administering aminoglycosides or vancomycin.^16^

The duration and severity of CSA-AKI are associated with worse outcomes.^21^^,^^29^^,^^30^ Patients who have a persistent CSA-AKI and/orsevere CSA-AKI (stage 2/3) have a longer length of stay in the ICU and hospital and increased morbidity and mortality.^10^^,^^21^^,^^31^ In the multivariable regression analysis, we demonstrate that in the EPIS-AKI dataset, certain factors are independently associated with the development of a moderate/severe CSA-AKI and/or persistent CSA-AKI. Most of these independent risk factors have already been described in previous studies with similar objectives.^13^^,^^32^^,^^33^ However, our results provide greater insight and differ from previous reports. For instance, a postoperative positive fluid balance until day 3 was identified as an independent risk factor for CSA-AKI. Furthermore, this study systematically demonstrates a connection between the use of the cell saver and an increased occurrence of postoperative AKI in a specific cohort. Given that our statistical model compared and sorted out all potential confounding factors (e.g. bleeding), we hypothesise that it might the application of the processed blood itself that contributes to the development of CSA-AKI. As the current evidence on this topic is often focused on CPB-associated haemolysis in general,^34^ or limited to sources with limited significance, such as case reports,^35^ this yields novel and clinically relevant insights.

The modifiable risk factors include the intraoperative use of aminoglycosides, postoperative haemodynamic instability, need for re-operation, and postoperative fluid balance until day 3 which are independently associated with all types of CSA-AKI. Even though a reverse causation might be possible (i.e. AKI from another cause resulting in toxic levels of the drug), avoidance of these risk factors and a raised awareness for markers of complications such as haemodynamic instability and need for re-operation may reduce the occurrence of CSA-AKI and modify its trajectory. Interventional trials targeting these factors have already proven beneficial for patient outcomes.^23^ An example for therapeutic adjustments has been demonstrated for aminoglycosides as potential nephrotoxins. In some situations, it might be possible to stop aminoglycosides and thus eliminate this risk factor. However, in situations in which aminoglycosides are indicated, a strict therapeutic drug monitoring should be used to minimise the nephrotoxic effects of aminoglycosides.

Our findings imply that there are several modifiable factors that can be optimised to reduce the occurrence of CSA-AKI. Implementing protocols that address these factors may positively affect the rate and trajectory of CSA-AKI.^26^^,^^27^ Moreover, our findings suggest that there are risk factors that are associated not only with the development of CSA-AKI but also with moderate/severe and persistent CSA-AKI. Our risk score appears to have good performance in predicting persistent CSA-AKI. Compared with previously described scores, such as the AKICS score,^14^ our score was developed based on a dataset of a multicentre study that included more than 160 centres and therefore reflects worldwide practice. In addition, our score might be easily used, as it requires fewer variables and utilises an AKI definition that is based on the KDIGO guidelines.^16^

A current limitation of our score is the lack of external validation. However, after further external validation, the score might be used to identify patients who have a high risk for developing persistent CSA-AKI. In addition, the score might be used in future studies for patient enrichment.

Our study has several strengths. First, the original EPIS-AKI study was a large international multicentre prospective observational study including a broad spectrum of patients from more than 160 centres who all had major surgery and were treated in an ICU after surgery. However, we acknowledge several limitations. This was a *post hoc* subgroup analysis. Thus, all analyses should be considered hypothesis-generating and do not allow for causal inference. Furthermore, although it is a multinational observational study, some regions only contributed a limited number of patients to this study, reducing the generalisability of the results.

### Conclusions

Overall, 25% of cardiac surgery patients developed cardiac surgery-associated acute kidney injury, a complication associated with significant morbidity. More than 33% of these patients experienced persistent cardiac surgery-associated acute kidney injury, which was associated with worst outcomes. Modifiable risk factors (e.g. nephrotoxic agents) were identified. Finally, a risk score for predicting persistent cardiac surgery-associated acute kidney injury was developed that, after further external validation, might be used to identify patients who have a high risk for developing persistent cardiac surgery-associated acute kidney injury.

## Authors’ contributions

Managed and performed the study: CS, FA, EB, AZ

Drafted the manuscript: CS, AZ

Designed the study: AZ

Critically revised the manuscript: DTE, RB

## Funding

Department of Anesthesiology, Intensive Care and Pain Medicine at the University Hospital Muenster (Muenster, Germany).

## Declarations of interest

AZ has received consulting fees from Astute-Biomerieux, Baxter, Bayer, Novartis, Guard Therapeutics, AM Pharma, Paion, Viatris, Renibus, Alexion, and Fresenius; research funding from Astute-Biomerieux, Fresenius, and Baxter; and speakers’ fees from Astute-Biomerieux, Fresenius, Baxter. The other authors declare that they have no conflicts of interest.
