# Supplementary material for: Risk factors, outcomes, and early prediction of cardiac surgery-associated acute kidney injury: a post hoc subgroup analysis of the Epidemiology of Surgery Associated Acute Kidney Injury study
Source: Br J Anaesth. 2025 Oct 9;136(1):34–42. doi: 10.1016/j.bja.2025.08.043 (PMC12851876; doi:10.1016/j.bja.2025.08.043)
Supplement: Multimedia component 1 [file mmc1.docx]

**Supplemental File: The EPIS-AKI Investigators**

[Alexander Zarboc](https://pubmed.ncbi.nlm.nih.gov/?term=%22Zarbock%20A%22%5BAuthor%5D)k, [Raphael Weis](https://pubmed.ncbi.nlm.nih.gov/?term=%22Weiss%20R%22%5BAuthor%5D)s, [Felix Albert](https://pubmed.ncbi.nlm.nih.gov/?term=%22Albert%20F%22%5BAuthor%5D), [Kristen Rutledge](https://pubmed.ncbi.nlm.nih.gov/?term=%22Rutledge%20K%22%5BAuthor%5D), [John A Kellum](https://pubmed.ncbi.nlm.nih.gov/?term=%22Kellum%20JA%22%5BAuthor%5D), [Rinaldo Bellomo](https://pubmed.ncbi.nlm.nih.gov/?term=%22Bellomo%20R%22%5BAuthor%5D), [Evgeny Grigoryev](https://pubmed.ncbi.nlm.nih.gov/?term=%22Grigoryev%20E%22%5BAuthor%5D), [Angel M Candela-Toha](https://pubmed.ncbi.nlm.nih.gov/?term=%22Candela-Toha%20AM%22%5BAuthor%5D), [Z Aslı Demir](https://pubmed.ncbi.nlm.nih.gov/?term=%22Demir%20ZA%22%5BAuthor%5D), [Vincent Legros](https://pubmed.ncbi.nlm.nih.gov/?term=%22Legros%20V%22%5BAuthor%5D), [Peter Rosenberger](https://pubmed.ncbi.nlm.nih.gov/?term=%22Rosenberger%20P%22%5BAuthor%5D), [Patricia Galán Menéndez](https://pubmed.ncbi.nlm.nih.gov/?term=%22Gal%C3%A1n%20Men%C3%A9ndez%20P%22%5BAuthor%5D), [Mercedes Garcia Alvarez](https://pubmed.ncbi.nlm.nih.gov/?term=%22Garcia%20Alvarez%20M%22%5BAuthor%5D), [Ke Peng](https://pubmed.ncbi.nlm.nih.gov/?term=%22Peng%20K%22%5BAuthor%5D), [Maxime Léger](https://pubmed.ncbi.nlm.nih.gov/?term=%22L%C3%A9ger%20M%22%5BAuthor%5D), [Wegdan Khalel](https://pubmed.ncbi.nlm.nih.gov/?term=%22Khalel%20W%22%5BAuthor%5D), [Mukadder Orhan-Sungur](https://pubmed.ncbi.nlm.nih.gov/?term=%22Orhan-Sungur%20M%22%5BAuthor%5D), [Melanie Meersch](https://pubmed.ncbi.nlm.nih.gov/?term=%22Meersch%20M%22%5BAuthor%5D), [Hichem Makhloufi](https://pubmed.ncbi.nlm.nih.gov/?term=%22Makhloufi%20H%22%5BAuthor%5D), [Rachida Sakhraoui](https://pubmed.ncbi.nlm.nih.gov/?term=%22Sakhraoui%20R%22%5BAuthor%5D), [Amel Ouyahia](https://pubmed.ncbi.nlm.nih.gov/?term=%22Ouyahia%20A%22%5BAuthor%5D), [Mounira Rais](https://pubmed.ncbi.nlm.nih.gov/?term=%22Rais%20M%22%5BAuthor%5D), [Aya Tinhinane Kouicem](https://pubmed.ncbi.nlm.nih.gov/?term=%22Kouicem%20AT%22%5BAuthor%5D), [Khawla Derwish](https://pubmed.ncbi.nlm.nih.gov/?term=%22Derwish%20K%22%5BAuthor%5D), [Meriem Abdoun](https://pubmed.ncbi.nlm.nih.gov/?term=%22Abdoun%20M%22%5BAuthor%5D), [Ilhem Ouahab](https://pubmed.ncbi.nlm.nih.gov/?term=%22Ouahab%20I%22%5BAuthor%5D), [Souad Bouaoud](https://pubmed.ncbi.nlm.nih.gov/?term=%22Bouaoud%20S%22%5BAuthor%5D), [Anisse Tidjane](https://pubmed.ncbi.nlm.nih.gov/?term=%22Tidjane%20A%22%5BAuthor%5D), [Carlos Jose Pérez Rivera](https://pubmed.ncbi.nlm.nih.gov/?term=%22Rivera%20CJP%22%5BAuthor%5D), [Juan Pablo García](https://pubmed.ncbi.nlm.nih.gov/?term=%22Garc%C3%ADa%20JP%22%5BAuthor%5D), [Fu-hai Ji](https://pubmed.ncbi.nlm.nih.gov/?term=%22Ji%20FH%22%5BAuthor%5D), [Zheng-min Ma](https://pubmed.ncbi.nlm.nih.gov/?term=%22Ma%20ZM%22%5BAuthor%5D), [Peter Sklienka](https://pubmed.ncbi.nlm.nih.gov/?term=%22Sklienka%20P%22%5BAuthor%5D), [Mohamed Gamal Elbahnasawy](https://pubmed.ncbi.nlm.nih.gov/?term=%22Elbahnasawy%20MG%22%5BAuthor%5D), [Shady Elsalhawy](https://pubmed.ncbi.nlm.nih.gov/?term=%22Elsalhawy%20S%22%5BAuthor%5D), [Ahmed Mahmoud Nafea](https://pubmed.ncbi.nlm.nih.gov/?term=%22Nafea%20AM%22%5BAuthor%5D), [Nermin A. Osman](https://pubmed.ncbi.nlm.nih.gov/?term=%22Osman%20NA%22%5BAuthor%5D), [Moataz Maher Emara](https://pubmed.ncbi.nlm.nih.gov/?term=%22Emara%20MM%22%5BAuthor%5D), [Mohamed Mamdouh Bonna](https://pubmed.ncbi.nlm.nih.gov/?term=%22Bonna%20MM%22%5BAuthor%5D), [Ibrahim Abdelmonaem Abdehaleem](https://pubmed.ncbi.nlm.nih.gov/?term=%22Abdehaleem%20IA%22%5BAuthor%5D), [Ahmed Mohamed Abbas](https://pubmed.ncbi.nlm.nih.gov/?term=%22Abbas%20AM%22%5BAuthor%5D), [Mostafa Samy Abbas](https://pubmed.ncbi.nlm.nih.gov/?term=%22Abbas%20MS%22%5BAuthor%5D), [Hany Mostafa Esmaeil](https://pubmed.ncbi.nlm.nih.gov/?term=%22Esmaeil%20HM%22%5BAuthor%5D), [Oliver Joannes-Boyau](https://pubmed.ncbi.nlm.nih.gov/?term=%22Joannes-Boyau%20O%22%5BAuthor%5D), [Thierry Floch](https://pubmed.ncbi.nlm.nih.gov/?term=%22Floch%20T%22%5BAuthor%5D), [Salvatore Muccio](https://pubmed.ncbi.nlm.nih.gov/?term=%22Muccio%20S%22%5BAuthor%5D), [Lison Menage-Innocenti](https://pubmed.ncbi.nlm.nih.gov/?term=%22Menage-Innocenti%20L%22%5BAuthor%5D), [Benjamin Brochet](https://pubmed.ncbi.nlm.nih.gov/?term=%22Brochet%20B%22%5BAuthor%5D), [Marion Leclercq-Rouget](https://pubmed.ncbi.nlm.nih.gov/?term=%22Leclercq-Rouget%20M%22%5BAuthor%5D), [Claire Geneve](https://pubmed.ncbi.nlm.nih.gov/?term=%22Geneve%20C%22%5BAuthor%5D), [Bernardita Valenzuela Mocarquer](https://pubmed.ncbi.nlm.nih.gov/?term=%22Mocarquer%20BV%22%5BAuthor%5D), [Christophe Aveline](https://pubmed.ncbi.nlm.nih.gov/?term=%22Aveline%20C%22%5BAuthor%5D), [Pierre Vautier](https://pubmed.ncbi.nlm.nih.gov/?term=%22Vautier%20P%22%5BAuthor%5D), [Julien Nadaud](https://pubmed.ncbi.nlm.nih.gov/?term=%22Nadaud%20J%22%5BAuthor%5D), [Thomas Rimmelé](https://pubmed.ncbi.nlm.nih.gov/?term=%22Rimmel%C3%A9%20T%22%5BAuthor%5D), [Valérie Cerro](https://pubmed.ncbi.nlm.nih.gov/?term=%22Cerro%20V%22%5BAuthor%5D), [Stéphanie Suria](https://pubmed.ncbi.nlm.nih.gov/?term=%22Suria%20S%22%5BAuthor%5D), [Jamie Elmawieh](https://pubmed.ncbi.nlm.nih.gov/?term=%22Elmawieh%20J%22%5BAuthor%5D), [Rita El-Jawiche](https://pubmed.ncbi.nlm.nih.gov/?term=%22El-Jawiche%20R%22%5BAuthor%5D), [Cédric Cirenei](https://pubmed.ncbi.nlm.nih.gov/?term=%22Cirenei%20C%22%5BAuthor%5D), [Gilles Lebuffe](https://pubmed.ncbi.nlm.nih.gov/?term=%22Lebuffe%20G%22%5BAuthor%5D), [Sébastien Ponsonnard](https://pubmed.ncbi.nlm.nih.gov/?term=%22Ponsonnard%20S%22%5BAuthor%5D), [Pierre-Yves Egreteau](https://pubmed.ncbi.nlm.nih.gov/?term=%22Egreteau%20PY%22%5BAuthor%5D), [Carole Ichai](https://pubmed.ncbi.nlm.nih.gov/?term=%22Ichai%20C%22%5BAuthor%5D), [Vanessa Jean-Michel](https://pubmed.ncbi.nlm.nih.gov/?term=%22Jean-Michel%20V%22%5BAuthor%5D), [Sigismond Lasocki](https://pubmed.ncbi.nlm.nih.gov/?term=%22Lasocki%20S%22%5BAuthor%5D), [Charline Masson](https://pubmed.ncbi.nlm.nih.gov/?term=%22Masson%20C%22%5BAuthor%5D), [Emmanuel Rineau](https://pubmed.ncbi.nlm.nih.gov/?term=%22Rineau%20E%22%5BAuthor%5D), [Viviane Cassisa](https://pubmed.ncbi.nlm.nih.gov/?term=%22Cassisa%20V%22%5BAuthor%5D), [Pierre Verrier](https://pubmed.ncbi.nlm.nih.gov/?term=%22Verrier%20P%22%5BAuthor%5D), [Enora Atchade](https://pubmed.ncbi.nlm.nih.gov/?term=%22Atchade%20E%22%5BAuthor%5D), [Charles-Edouard Rochon](https://pubmed.ncbi.nlm.nih.gov/?term=%22Rochon%20CE%22%5BAuthor%5D), [Vidal Quentin](https://pubmed.ncbi.nlm.nih.gov/?term=%22Quentin%20V%22%5BAuthor%5D), [Nina Queixalos](https://pubmed.ncbi.nlm.nih.gov/?term=%22Queixalos%20N%22%5BAuthor%5D), [Thierry Braun](https://pubmed.ncbi.nlm.nih.gov/?term=%22Braun%20T%22%5BAuthor%5D), [Hubert Grand](https://pubmed.ncbi.nlm.nih.gov/?term=%22Grand%20H%22%5BAuthor%5D), [Nicolas Mayeur](https://pubmed.ncbi.nlm.nih.gov/?term=%22Mayeur%20N%22%5BAuthor%5D), [Marie Pasquie](https://pubmed.ncbi.nlm.nih.gov/?term=%22Pasquie%20M%22%5BAuthor%5D), [Pierre Garçon](https://pubmed.ncbi.nlm.nih.gov/?term=%22Gar%C3%A7on%20P%22%5BAuthor%5D), [Vincent Bruckert](https://pubmed.ncbi.nlm.nih.gov/?term=%22Bruckert%20V%22%5BAuthor%5D), [Gaël Pradel](https://pubmed.ncbi.nlm.nih.gov/?term=%22Pradel%20G%22%5BAuthor%5D), [Andersen Ramorasata](https://pubmed.ncbi.nlm.nih.gov/?term=%22Ramorasata%20A%22%5BAuthor%5D), [Céline Ravry](https://pubmed.ncbi.nlm.nih.gov/?term=%22Ravry%20C%22%5BAuthor%5D), [Nicolas Mottard](https://pubmed.ncbi.nlm.nih.gov/?term=%22Mottard%20N%22%5BAuthor%5D), [Thilo von Groote](https://pubmed.ncbi.nlm.nih.gov/?term=%22von%20Groote%20T%22%5BAuthor%5D), [Christian Dörr](https://pubmed.ncbi.nlm.nih.gov/?term=%22D%C3%B6rr%20C%22%5BAuthor%5D), [Mira Küllmar](https://pubmed.ncbi.nlm.nih.gov/?term=%22K%C3%BCllmar%20M%22%5BAuthor%5D), [Christina Massoth](https://pubmed.ncbi.nlm.nih.gov/?term=%22Massoth%20C%22%5BAuthor%5D), [Arash Motekallemi](https://pubmed.ncbi.nlm.nih.gov/?term=%22Motekallemi%20A%22%5BAuthor%5D), [Khaschayar Saadat-Gilani](https://pubmed.ncbi.nlm.nih.gov/?term=%22Saadat-Gilani%20K%22%5BAuthor%5D), [Laura Kerschke](https://pubmed.ncbi.nlm.nih.gov/?term=%22Kerschke%20L%22%5BAuthor%5D), [Michael Storck](https://pubmed.ncbi.nlm.nih.gov/?term=%22Storck%20M%22%5BAuthor%5D), [Julian Varghese](https://pubmed.ncbi.nlm.nih.gov/?term=%22Varghese%20J%22%5BAuthor%5D), [Carola Wempe](https://pubmed.ncbi.nlm.nih.gov/?term=%22Wempe%20C%22%5BAuthor%5D), [Linda Grüßer](https://pubmed.ncbi.nlm.nih.gov/?term=%22Gr%C3%BC%C3%9Fer%20L%22%5BAuthor%5D), [Ana Kowark](https://pubmed.ncbi.nlm.nih.gov/?term=%22Kowark%20A%22%5BAuthor%5D), [Timo Brandenburger](https://pubmed.ncbi.nlm.nih.gov/?term=%22Brandenburger%20T%22%5BAuthor%5D), [Andreas Hohn](https://pubmed.ncbi.nlm.nih.gov/?term=%22Hohn%20A%22%5BAuthor%5D), [Helene Häberle](https://pubmed.ncbi.nlm.nih.gov/?term=%22H%C3%A4berle%20H%22%5BAuthor%5D), [Pascal Hofmann](https://pubmed.ncbi.nlm.nih.gov/?term=%22Hofmann%20P%22%5BAuthor%5D), [Jonathan Kuhle](https://pubmed.ncbi.nlm.nih.gov/?term=%22Kuhle%20J%22%5BAuthor%5D), [Stefanie Calov](https://pubmed.ncbi.nlm.nih.gov/?term=%22Calov%20S%22%5BAuthor%5D), [Alice Marie Bernard](https://pubmed.ncbi.nlm.nih.gov/?term=%22Bernard%20AM%22%5BAuthor%5D), [Valbona Mirakaj](https://pubmed.ncbi.nlm.nih.gov/?term=%22Mirakaj%20V%22%5BAuthor%5D), [Kathrin Weber](https://pubmed.ncbi.nlm.nih.gov/?term=%22Weber%20K%22%5BAuthor%5D), [Kathrin Pfister](https://pubmed.ncbi.nlm.nih.gov/?term=%22Pfister%20K%22%5BAuthor%5D), [Lena Stetz](https://pubmed.ncbi.nlm.nih.gov/?term=%22Stetz%20L%22%5BAuthor%5D), [Sarah Dorothea Müller](https://pubmed.ncbi.nlm.nih.gov/?term=%22M%C3%BCller%20SD%22%5BAuthor%5D), [Stephan Klaus](https://pubmed.ncbi.nlm.nih.gov/?term=%22Klaus%20S%22%5BAuthor%5D), [Marco Sadlo](https://pubmed.ncbi.nlm.nih.gov/?term=%22Sadlo%20M%22%5BAuthor%5D), [Carina-Kristin Stenger](https://pubmed.ncbi.nlm.nih.gov/?term=%22Stenger%20CK%22%5BAuthor%5D), [Ulrich Göbel](https://pubmed.ncbi.nlm.nih.gov/?term=%22G%C3%B6bel%20U%22%5BAuthor%5D), [Matthias Heringlake](https://pubmed.ncbi.nlm.nih.gov/?term=%22Heringlake%20M%22%5BAuthor%5D), [Eleni Arnaoutoglou](https://pubmed.ncbi.nlm.nih.gov/?term=%22Arnaoutoglou%20E%22%5BAuthor%5D), [Panagiota Stratigopoulou](https://pubmed.ncbi.nlm.nih.gov/?term=%22Stratigopoulou%20P%22%5BAuthor%5D), [Pantazi Danai](https://pubmed.ncbi.nlm.nih.gov/?term=%22Danai%20P%22%5BAuthor%5D), [Antonia Dimakopoulou](https://pubmed.ncbi.nlm.nih.gov/?term=%22Dimakopoulou%20A%22%5BAuthor%5D), [Apostolos-Alkiviadis Menis](https://pubmed.ncbi.nlm.nih.gov/?term=%22Menis%20AA%22%5BAuthor%5D), [Orestis Ioannidis](https://pubmed.ncbi.nlm.nih.gov/?term=%22Ioannidis%20O%22%5BAuthor%5D), [Humam Jalaawiy](https://pubmed.ncbi.nlm.nih.gov/?term=%22Jalaawiy%20H%22%5BAuthor%5D), [Aeshah Anwar](https://pubmed.ncbi.nlm.nih.gov/?term=%22Anwar%20A%22%5BAuthor%5D), [Hashim Talib Hashim](https://pubmed.ncbi.nlm.nih.gov/?term=%22Hashim%20HT%22%5BAuthor%5D), [Hogir Imad Rasheed Aldawoody](https://pubmed.ncbi.nlm.nih.gov/?term=%22Aldawoody%20HIR%22%5BAuthor%5D), [Andrea Cortegiani](https://pubmed.ncbi.nlm.nih.gov/?term=%22Cortegiani%20A%22%5BAuthor%5D), [Mariachiara Ippolito](https://pubmed.ncbi.nlm.nih.gov/?term=%22Ippolito%20M%22%5BAuthor%5D), [Claudia Marino](https://pubmed.ncbi.nlm.nih.gov/?term=%22Marino%20C%22%5BAuthor%5D), [Gabriele Presti](https://pubmed.ncbi.nlm.nih.gov/?term=%22Presti%20G%22%5BAuthor%5D), [Dario Calogero Fricano](https://pubmed.ncbi.nlm.nih.gov/?term=%22Fricano%20DC%22%5BAuthor%5D), [Silvia De Rosa](https://pubmed.ncbi.nlm.nih.gov/?term=%22De%20Rosa%20S%22%5BAuthor%5D), [Andrea Bianchin](https://pubmed.ncbi.nlm.nih.gov/?term=%22Bianchin%20A%22%5BAuthor%5D), [Gianluca Paternoster](https://pubmed.ncbi.nlm.nih.gov/?term=%22Paternoster%20G%22%5BAuthor%5D), [Umberto Fasciano](https://pubmed.ncbi.nlm.nih.gov/?term=%22Fasciano%20U%22%5BAuthor%5D), [Salvatore Lucio Cutuli](https://pubmed.ncbi.nlm.nih.gov/?term=%22Cutuli%20SL%22%5BAuthor%5D), [Savino Spadaro](https://pubmed.ncbi.nlm.nih.gov/?term=%22Spadaro%20S%22%5BAuthor%5D), [Enrico Bussolati](https://pubmed.ncbi.nlm.nih.gov/?term=%22Bussolati%20E%22%5BAuthor%5D), [Marco Palmieri](https://pubmed.ncbi.nlm.nih.gov/?term=%22Palmieri%20M%22%5BAuthor%5D), [Carlo Alberto Volta](https://pubmed.ncbi.nlm.nih.gov/?term=%22Volta%20CA%22%5BAuthor%5D), [Vincenzo Francesco Tripodi](https://pubmed.ncbi.nlm.nih.gov/?term=%22Tripodi%20VF%22%5BAuthor%5D), [Diego Fiume](https://pubmed.ncbi.nlm.nih.gov/?term=%22Fiume%20D%22%5BAuthor%5D), [Angela Iuorio](https://pubmed.ncbi.nlm.nih.gov/?term=%22Iuorio%20A%22%5BAuthor%5D), [Clemente Santorsola](https://pubmed.ncbi.nlm.nih.gov/?term=%22Santorsola%20C%22%5BAuthor%5D), [Bilal Abu-Hussein](https://pubmed.ncbi.nlm.nih.gov/?term=%22Abu-Hussein%20B%22%5BAuthor%5D), [Khaled Hasanein](https://pubmed.ncbi.nlm.nih.gov/?term=%22Hasanein%20K%22%5BAuthor%5D), [Seokyung Shin](https://pubmed.ncbi.nlm.nih.gov/?term=%22Shin%20S%22%5BAuthor%5D), [Jongyoon Baek](https://pubmed.ncbi.nlm.nih.gov/?term=%22Baek%20J%22%5BAuthor%5D), [Sehui Kim](https://pubmed.ncbi.nlm.nih.gov/?term=%22Kim%20S%22%5BAuthor%5D), [Muhammed Elhadi](https://pubmed.ncbi.nlm.nih.gov/?term=%22Elhadi%20M%22%5BAuthor%5D), [Wafa Aldressi](https://pubmed.ncbi.nlm.nih.gov/?term=%22Aldressi%20W%22%5BAuthor%5D), [Issa A. Abuzeid](https://pubmed.ncbi.nlm.nih.gov/?term=%22Abuzeid%20IA%22%5BAuthor%5D), [Mohammed N. Albaraesi](https://pubmed.ncbi.nlm.nih.gov/?term=%22Albaraesi%20MN%22%5BAuthor%5D), [Mohamed Aziz Moftah](https://pubmed.ncbi.nlm.nih.gov/?term=%22Moftah%20MA%22%5BAuthor%5D), [Sarah Aldressi](https://pubmed.ncbi.nlm.nih.gov/?term=%22Aldressi%20S%22%5BAuthor%5D), [Eman Abdulwahed](https://pubmed.ncbi.nlm.nih.gov/?term=%22Abdulwahed%20E%22%5BAuthor%5D), [Entisar Ahmed Ali Alshareea](https://pubmed.ncbi.nlm.nih.gov/?term=%22Alshareea%20EAA%22%5BAuthor%5D), [Akram Abdulhamid Ashur Abujrad](https://pubmed.ncbi.nlm.nih.gov/?term=%22Abujrad%20AAA%22%5BAuthor%5D), [Reem Ghmagh](https://pubmed.ncbi.nlm.nih.gov/?term=%22Ghmagh%20R%22%5BAuthor%5D), [Marwa Isa Biala](https://pubmed.ncbi.nlm.nih.gov/?term=%22Biala%20MI%22%5BAuthor%5D), [Rayet Al Islam Benjouira](https://pubmed.ncbi.nlm.nih.gov/?term=%22Al%20Islam%20Benjouira%20R%22%5BAuthor%5D), [Mohamed Aliwa](https://pubmed.ncbi.nlm.nih.gov/?term=%22Aliwa%20M%22%5BAuthor%5D), [Ahmed Msherghi](https://pubmed.ncbi.nlm.nih.gov/?term=%22Msherghi%20A%22%5BAuthor%5D), [Ahmed Tuwaib](https://pubmed.ncbi.nlm.nih.gov/?term=%22Tuwaib%20A%22%5BAuthor%5D), [Tahani Mustafa](https://pubmed.ncbi.nlm.nih.gov/?term=%22Mustafa%20T%22%5BAuthor%5D), [Haifa Zriba](https://pubmed.ncbi.nlm.nih.gov/?term=%22Zriba%20H%22%5BAuthor%5D), [Hamza Mahmoud Agilla](https://pubmed.ncbi.nlm.nih.gov/?term=%22Agilla%20HM%22%5BAuthor%5D), [Bahaeddin Taher Sadek Ben Hamida](https://pubmed.ncbi.nlm.nih.gov/?term=%22Hamida%20BTSB%22%5BAuthor%5D), [Rema Hassan Mohamed Otman](https://pubmed.ncbi.nlm.nih.gov/?term=%22Otman%20RHM%22%5BAuthor%5D), [Maja Mojsova Mijovska](https://pubmed.ncbi.nlm.nih.gov/?term=%22Mijovska%20MM%22%5BAuthor%5D), [Anne Marie Camilleri Podesta](https://pubmed.ncbi.nlm.nih.gov/?term=%22Podesta%20AMC%22%5BAuthor%5D), [Gilberto Adrián Gasca López](https://pubmed.ncbi.nlm.nih.gov/?term=%22L%C3%B3pez%20GAG%22%5BAuthor%5D), [Sarah Amro](https://pubmed.ncbi.nlm.nih.gov/?term=%22Amro%20S%22%5BAuthor%5D), [Rita de Freitas Regufe](https://pubmed.ncbi.nlm.nih.gov/?term=%22de%20Freitas%20Regufe%20R%22%5BAuthor%5D), [Artem Ivkin](https://pubmed.ncbi.nlm.nih.gov/?term=%22Ivkin%20A%22%5BAuthor%5D), [Dmitriy Balakhnin](https://pubmed.ncbi.nlm.nih.gov/?term=%22Balakhnin%20D%22%5BAuthor%5D), [Dmitriy Shukevich](https://pubmed.ncbi.nlm.nih.gov/?term=%22Shukevich%20D%22%5BAuthor%5D), [Michael Yaroustovsky](https://pubmed.ncbi.nlm.nih.gov/?term=%22Yaroustovsky%20M%22%5BAuthor%5D), [Abdulnaser Barmou](https://pubmed.ncbi.nlm.nih.gov/?term=%22Barmou%20A%22%5BAuthor%5D), [Alexander Kaserer](https://pubmed.ncbi.nlm.nih.gov/?term=%22Kaserer%20A%22%5BAuthor%5D), [Clara Castellucci](https://pubmed.ncbi.nlm.nih.gov/?term=%22Castellucci%20C%22%5BAuthor%5D), [Samira Akbas](https://pubmed.ncbi.nlm.nih.gov/?term=%22Akbas%20S%22%5BAuthor%5D), [Andreja Möller Petrun](https://pubmed.ncbi.nlm.nih.gov/?term=%22Petrun%20AM%22%5BAuthor%5D), [Irena Gregorcic](https://pubmed.ncbi.nlm.nih.gov/?term=%22Gregorcic%20I%22%5BAuthor%5D), [Vesna Sok](https://pubmed.ncbi.nlm.nih.gov/?term=%22Sok%20V%22%5BAuthor%5D), [Andre Links](https://pubmed.ncbi.nlm.nih.gov/?term=%22Links%20A%22%5BAuthor%5D), [Elizabeth Bárcena Barreto](https://pubmed.ncbi.nlm.nih.gov/?term=%22Barreto%20EB%22%5BAuthor%5D), [Javier Ripollés Melchor](https://pubmed.ncbi.nlm.nih.gov/?term=%22Melchor%20JR%22%5BAuthor%5D), [Ángel Becerra-Bolaños](https://pubmed.ncbi.nlm.nih.gov/?term=%22Becerra-Bola%C3%B1os%20%C3%81%22%5BAuthor%5D), [Aurelio Rodríguez-Pérez](https://pubmed.ncbi.nlm.nih.gov/?term=%22Rodr%C3%ADguez-P%C3%A9rez%20A%22%5BAuthor%5D), [Javier Mata Estévez](https://pubmed.ncbi.nlm.nih.gov/?term=%22Est%C3%A9vez%20JM%22%5BAuthor%5D), [Juan Mulet Matas](https://pubmed.ncbi.nlm.nih.gov/?term=%22Matas%20JM%22%5BAuthor%5D), [Sara Pérez Palao](https://pubmed.ncbi.nlm.nih.gov/?term=%22Palao%20SP%22%5BAuthor%5D), [Mercedes García Álvarez](https://pubmed.ncbi.nlm.nih.gov/?term=%22%C3%81lvarez%20MG%22%5BAuthor%5D), [Albert Bainac Albadalejo](https://pubmed.ncbi.nlm.nih.gov/?term=%22Albadalejo%20AB%22%5BAuthor%5D), [Astrid Batalla González](https://pubmed.ncbi.nlm.nih.gov/?term=%22Gonz%C3%A1lez%20AB%22%5BAuthor%5D), [Ana María Gómez Caro](https://pubmed.ncbi.nlm.nih.gov/?term=%22Caro%20AMG%22%5BAuthor%5D), [Ignacio Hinojal Blanco](https://pubmed.ncbi.nlm.nih.gov/?term=%22Blanco%20IH%22%5BAuthor%5D), [Diego Toral Fernandez](https://pubmed.ncbi.nlm.nih.gov/?term=%22Fernandez%20DT%22%5BAuthor%5D), [Gracia Herranz Perez](https://pubmed.ncbi.nlm.nih.gov/?term=%22Perez%20GH%22%5BAuthor%5D), [Margarita Logroño Ejea](https://pubmed.ncbi.nlm.nih.gov/?term=%22Ejea%20ML%22%5BAuthor%5D), [Noelia de la Rosa Ruiz](https://pubmed.ncbi.nlm.nih.gov/?term=%22de%20la%20Rosa%20Ruiz%20N%22%5BAuthor%5D), [María Gastaca Abasolo](https://pubmed.ncbi.nlm.nih.gov/?term=%22Abasolo%20MG%22%5BAuthor%5D), [Lourdes Ferreira](https://pubmed.ncbi.nlm.nih.gov/?term=%22Ferreira%20L%22%5BAuthor%5D), [Félix Lobato](https://pubmed.ncbi.nlm.nih.gov/?term=%22Lobato%20F%22%5BAuthor%5D), [Marta Aguado Sevilla](https://pubmed.ncbi.nlm.nih.gov/?term=%22Sevilla%20MA%22%5BAuthor%5D), [Andres Erazo](https://pubmed.ncbi.nlm.nih.gov/?term=%22Erazo%20A%22%5BAuthor%5D), [Berta Castellano Paulis](https://pubmed.ncbi.nlm.nih.gov/?term=%22Paulis%20BC%22%5BAuthor%5D), [Isabel de la Calle Gil](https://pubmed.ncbi.nlm.nih.gov/?term=%22de%20la%20Calle%20Gil%20I%22%5BAuthor%5D), [Peter Adamove](https://pubmed.ncbi.nlm.nih.gov/?term=%22Adamove%20P%22%5BAuthor%5D), [Francho Miguel Blasco Blasco](https://pubmed.ncbi.nlm.nih.gov/?term=%22Blasco%20Blasco%20FM%22%5BAuthor%5D), [Jose Ignacio García-Sánchez](https://pubmed.ncbi.nlm.nih.gov/?term=%22Garc%C3%ADa-S%C3%A1nchez%20JI%22%5BAuthor%5D), [Sara García Zamorano](https://pubmed.ncbi.nlm.nih.gov/?term=%22Zamorano%20SG%22%5BAuthor%5D), [Natalia Gijón Herreros](https://pubmed.ncbi.nlm.nih.gov/?term=%22Herreros%20NG%22%5BAuthor%5D), [Raquel Callejas](https://pubmed.ncbi.nlm.nih.gov/?term=%22Callejas%20R%22%5BAuthor%5D), [Mercedes Estaire Gómez](https://pubmed.ncbi.nlm.nih.gov/?term=%22G%C3%B3mez%20ME%22%5BAuthor%5D), [Elisabeth Claros-Llamas](https://pubmed.ncbi.nlm.nih.gov/?term=%22Claros-Llamas%20E%22%5BAuthor%5D), [Pilar Cobeta-Orduña](https://pubmed.ncbi.nlm.nih.gov/?term=%22Cobeta-Ordu%C3%B1a%20P%22%5BAuthor%5D), [Pascual Crespo-Aliseda](https://pubmed.ncbi.nlm.nih.gov/?term=%22Crespo-Aliseda%20P%22%5BAuthor%5D), [Trinidad Dorado-Díaz](https://pubmed.ncbi.nlm.nih.gov/?term=%22Dorado-D%C3%ADaz%20T%22%5BAuthor%5D), [María Gómez-Rojo](https://pubmed.ncbi.nlm.nih.gov/?term=%22G%C3%B3mez-Rojo%20M%22%5BAuthor%5D), [M. Nuria Mané-Ruiz](https://pubmed.ncbi.nlm.nih.gov/?term=%22Man%C3%A9-Ruiz%20MN%22%5BAuthor%5D), [M. Carmen Martín-González](https://pubmed.ncbi.nlm.nih.gov/?term=%22Mart%C3%ADn-Gonz%C3%A1lez%20MC%22%5BAuthor%5D), [Adolfo Martínez-Pérez](https://pubmed.ncbi.nlm.nih.gov/?term=%22Mart%C3%ADnez-P%C3%A9rez%20A%22%5BAuthor%5D), [Carlos Tiscar](https://pubmed.ncbi.nlm.nih.gov/?term=%22Tiscar%20C%22%5BAuthor%5D), [Verónica Estepa Calvo](https://pubmed.ncbi.nlm.nih.gov/?term=%22Calvo%20VE%22%5BAuthor%5D), [Laura Llinares Espí](https://pubmed.ncbi.nlm.nih.gov/?term=%22Esp%C3%AD%20LL%22%5BAuthor%5D), [Yuri Santiago Loaiza Aldeán](https://pubmed.ncbi.nlm.nih.gov/?term=%22Alde%C3%A1n%20YSL%22%5BAuthor%5D), [Víctor Morales Ariza](https://pubmed.ncbi.nlm.nih.gov/?term=%22Ariza%20VM%22%5BAuthor%5D), [Laura Villarino Vila](https://pubmed.ncbi.nlm.nih.gov/?term=%22Vila%20LV%22%5BAuthor%5D), [Francisco Javier García-Miguel](https://pubmed.ncbi.nlm.nih.gov/?term=%22Garc%C3%ADa-Miguel%20FJ%22%5BAuthor%5D), [Elfayadh S. M. Suliman](https://pubmed.ncbi.nlm.nih.gov/?term=%22Suliman%20ESM%22%5BAuthor%5D), [Ahmed Mohamed Ibrahim](https://pubmed.ncbi.nlm.nih.gov/?term=%22Ibrahim%20AM%22%5BAuthor%5D), [Hammad Ali Fadlalmola](https://pubmed.ncbi.nlm.nih.gov/?term=%22Fadlalmola%20HA%22%5BAuthor%5D), [Sarya Swed](https://pubmed.ncbi.nlm.nih.gov/?term=%22Swed%20S%22%5BAuthor%5D), [Vin-Cent Wu](https://pubmed.ncbi.nlm.nih.gov/?term=%22Wu%20VC%22%5BAuthor%5D), [Demet Altun](https://pubmed.ncbi.nlm.nih.gov/?term=%22Altun%20D%22%5BAuthor%5D), [Nur Canbolat](https://pubmed.ncbi.nlm.nih.gov/?term=%22Canbolat%20N%22%5BAuthor%5D), [Müşerref Beril Dinçer](https://pubmed.ncbi.nlm.nih.gov/?term=%22Din%C3%A7er%20MB%22%5BAuthor%5D), [Serap Aktas Yildirim](https://pubmed.ncbi.nlm.nih.gov/?term=%22Yildirim%20SA%22%5BAuthor%5D), [Muzeyyen Iyigun](https://pubmed.ncbi.nlm.nih.gov/?term=%22Iyigun%20M%22%5BAuthor%5D), [Davud Yapıcı](https://pubmed.ncbi.nlm.nih.gov/?term=%22Yap%C4%B1c%C4%B1%20D%22%5BAuthor%5D), [Levent Özdemir](https://pubmed.ncbi.nlm.nih.gov/?term=%22%C3%96zdemir%20L%22%5BAuthor%5D), [Aslınur Sagün](https://pubmed.ncbi.nlm.nih.gov/?term=%22Sag%C3%BCn%20A%22%5BAuthor%5D), [Neval Boztug](https://pubmed.ncbi.nlm.nih.gov/?term=%22Boztug%20N%22%5BAuthor%5D), [Emel Gündüz](https://pubmed.ncbi.nlm.nih.gov/?term=%22G%C3%BCnd%C3%BCz%20E%22%5BAuthor%5D), [Demet Lafli-Tunay](https://pubmed.ncbi.nlm.nih.gov/?term=%22Lafli-Tunay%20D%22%5BAuthor%5D), [Deniz Karakaya](https://pubmed.ncbi.nlm.nih.gov/?term=%22Karakaya%20D%22%5BAuthor%5D), [Burhan Dost](https://pubmed.ncbi.nlm.nih.gov/?term=%22Dost%20B%22%5BAuthor%5D), [Ozgur Komurcu](https://pubmed.ncbi.nlm.nih.gov/?term=%22Komurcu%20O%22%5BAuthor%5D), [Ozlem Korkmaz Dilmen](https://pubmed.ncbi.nlm.nih.gov/?term=%22Dilmen%20OK%22%5BAuthor%5D), [Eren Fatma Akcil](https://pubmed.ncbi.nlm.nih.gov/?term=%22Akcil%20EF%22%5BAuthor%5D), [Yusuf Tunali](https://pubmed.ncbi.nlm.nih.gov/?term=%22Tunali%20Y%22%5BAuthor%5D), [Gulay Ok](https://pubmed.ncbi.nlm.nih.gov/?term=%22Ok%20G%22%5BAuthor%5D), [Eda Tok-Alsina](https://pubmed.ncbi.nlm.nih.gov/?term=%22Tok-Alsina%20E%22%5BAuthor%5D), [Cengiz Polat](https://pubmed.ncbi.nlm.nih.gov/?term=%22Polat%20C%22%5BAuthor%5D), [Nurcan Kızılcık](https://pubmed.ncbi.nlm.nih.gov/?term=%22K%C4%B1z%C4%B1lc%C4%B1k%20N%22%5BAuthor%5D), [Öznur Şen](https://pubmed.ncbi.nlm.nih.gov/?term=%22%C5%9Een%20%C3%96%22%5BAuthor%5D), [Kamil Darçın](https://pubmed.ncbi.nlm.nih.gov/?term=%22Dar%C3%A7%C4%B1n%20K%22%5BAuthor%5D), [Semra Uğur](https://pubmed.ncbi.nlm.nih.gov/?term=%22U%C4%9Fur%20S%22%5BAuthor%5D), [Yavuz Gürkan](https://pubmed.ncbi.nlm.nih.gov/?term=%22G%C3%BCrkan%20Y%22%5BAuthor%5D), [Kemal Tolga Saracoglu](https://pubmed.ncbi.nlm.nih.gov/?term=%22Saracoglu%20KT%22%5BAuthor%5D), [Özge Yıldız-Koyuncu](https://pubmed.ncbi.nlm.nih.gov/?term=%22Y%C4%B1ld%C4%B1z-Koyuncu%20%C3%96%22%5BAuthor%5D), [Z. Aslı Demir](https://pubmed.ncbi.nlm.nih.gov/?term=%22Demir%20ZA%22%5BAuthor%5D), [N. Aysun Postacı](https://pubmed.ncbi.nlm.nih.gov/?term=%22Postac%C4%B1%20NA%22%5BAuthor%5D), [Ayşegül Özgök](https://pubmed.ncbi.nlm.nih.gov/?term=%22%C3%96zg%C3%B6k%20A%22%5BAuthor%5D), [Ümit Karadeniz](https://pubmed.ncbi.nlm.nih.gov/?term=%22Karadeniz%20%C3%9C%22%5BAuthor%5D), [Hülya Yiğit Özay](https://pubmed.ncbi.nlm.nih.gov/?term=%22%C3%96zay%20HY%22%5BAuthor%5D), [Eda Balcı](https://pubmed.ncbi.nlm.nih.gov/?term=%22Balc%C4%B1%20E%22%5BAuthor%5D), [Nevriye Salman](https://pubmed.ncbi.nlm.nih.gov/?term=%22Salman%20N%22%5BAuthor%5D), [Behiç Girgin](https://pubmed.ncbi.nlm.nih.gov/?term=%22Girgin%20B%22%5BAuthor%5D), [Ozlem Sagir](https://pubmed.ncbi.nlm.nih.gov/?term=%22Sagir%20O%22%5BAuthor%5D), [Hafize Fisun Demir](https://pubmed.ncbi.nlm.nih.gov/?term=%22Demir%20HF%22%5BAuthor%5D), [Fatih Ugun](https://pubmed.ncbi.nlm.nih.gov/?term=%22Ugun%20F%22%5BAuthor%5D), [Hüseyin İlksen Toprak](https://pubmed.ncbi.nlm.nih.gov/?term=%22Toprak%20H%C4%B0%22%5BAuthor%5D), [Mustafa Soner Özcan](https://pubmed.ncbi.nlm.nih.gov/?term=%22%C3%96zcan%20MS%22%5BAuthor%5D), [Filiz Alkaya-Solmaz](https://pubmed.ncbi.nlm.nih.gov/?term=%22Alkaya-Solmaz%20F%22%5BAuthor%5D), [Mehmet Yilmaz](https://pubmed.ncbi.nlm.nih.gov/?term=%22Yilmaz%20M%22%5BAuthor%5D), [Umran Karaca](https://pubmed.ncbi.nlm.nih.gov/?term=%22Karaca%20U%22%5BAuthor%5D), [Sevtap Hekimoglu Şahin](https://pubmed.ncbi.nlm.nih.gov/?term=%22%C5%9Eahin%20SH%22%5BAuthor%5D), [Süheyla Karadağ Erkoç](https://pubmed.ncbi.nlm.nih.gov/?term=%22Erko%C3%A7%20SK%22%5BAuthor%5D), [Neslihan Alkış](https://pubmed.ncbi.nlm.nih.gov/?term=%22Alk%C4%B1%C5%9F%20N%22%5BAuthor%5D), [Volkan Baytaş](https://pubmed.ncbi.nlm.nih.gov/?term=%22Bayta%C5%9F%20V%22%5BAuthor%5D), [Engin Erturk](https://pubmed.ncbi.nlm.nih.gov/?term=%22Erturk%20E%22%5BAuthor%5D), [Sedat Saylan](https://pubmed.ncbi.nlm.nih.gov/?term=%22Saylan%20S%22%5BAuthor%5D), [Ali Akdogan](https://pubmed.ncbi.nlm.nih.gov/?term=%22Akdogan%20A%22%5BAuthor%5D), [Beyza Büyükgebiz Yeşil](https://pubmed.ncbi.nlm.nih.gov/?term=%22Ye%C5%9Fil%20BB%22%5BAuthor%5D), [Omer Faruk Boran](https://pubmed.ncbi.nlm.nih.gov/?term=%22Boran%20OF%22%5BAuthor%5D), [Yavuz Orak](https://pubmed.ncbi.nlm.nih.gov/?term=%22Orak%20Y%22%5BAuthor%5D), [Feyza Çalişir](https://pubmed.ncbi.nlm.nih.gov/?term=%22%C3%87ali%C5%9Fir%20F%22%5BAuthor%5D), [Sibel Büyükçoban](https://pubmed.ncbi.nlm.nih.gov/?term=%22B%C3%BCy%C3%BCk%C3%A7oban%20S%22%5BAuthor%5D), [Bahar Kuvaki](https://pubmed.ncbi.nlm.nih.gov/?term=%22Kuvaki%20B%22%5BAuthor%5D), [Seda Cansabuncu](https://pubmed.ncbi.nlm.nih.gov/?term=%22Cansabuncu%20S%22%5BAuthor%5D), [Selcan Akesen](https://pubmed.ncbi.nlm.nih.gov/?term=%22Akesen%20S%22%5BAuthor%5D), [Suna Gören](https://pubmed.ncbi.nlm.nih.gov/?term=%22G%C3%B6ren%20S%22%5BAuthor%5D), [Tugce Yeniocak](https://pubmed.ncbi.nlm.nih.gov/?term=%22Yeniocak%20T%22%5BAuthor%5D), [Osman Orman](https://pubmed.ncbi.nlm.nih.gov/?term=%22Orman%20O%22%5BAuthor%5D), [Özlem Ersoy Karka](https://pubmed.ncbi.nlm.nih.gov/?term=%22Karka%20%C3%96E%22%5BAuthor%5D), [Tulay Sahin](https://pubmed.ncbi.nlm.nih.gov/?term=%22Sahin%20T%22%5BAuthor%5D), [Natalia Momot](https://pubmed.ncbi.nlm.nih.gov/?term=%22Momot%20N%22%5BAuthor%5D), [Anna Panchenko](https://pubmed.ncbi.nlm.nih.gov/?term=%22Panchenko%20A%22%5BAuthor%5D), [Jean-Francois Pittet](https://pubmed.ncbi.nlm.nih.gov/?term=%22Pittet%20JF%22%5BAuthor%5D), [Ahmed Albishti](https://pubmed.ncbi.nlm.nih.gov/?term=%22Albishti%20A%22%5BAuthor%5D), [Mohamed Alsori](https://pubmed.ncbi.nlm.nih.gov/?term=%22Alsori%20M%22%5BAuthor%5D), and [Salmi Hamza Abrayik](https://pubmed.ncbi.nlm.nih.gov/?term=%22Abrayik%20SH%22%5BAuthor%5D)
