# Supplementary material for: Risk factors, outcomes, and early prediction of cardiac surgery-associated acute kidney injury: a post hoc subgroup analysis of the Epidemiology of Surgery Associated Acute Kidney Injury study
Source: Br J Anaesth. 2025 Oct 9;136(1):34–42. doi: 10.1016/j.bja.2025.08.043 (PMC12851876; doi:10.1016/j.bja.2025.08.043)
Supplement: Multimedia component 2 [file mmc2.docx]

**Supplementary Appendix**

**Methods and statistical analysis**

Multivariable logistic regression analyses were performed to identify and assess the association of other risk factors for binary outcomes, i.e. CSA-AKI, moderate/severe CSA-AKI, persistent CSA-AKI and hospital mortality. In a first step, we selected variables that, according to previous clinical knowledge, could be associated with one or more of these outcomes: baseline variables (age, sex, body mass index (BMI), serum-creatinine prior to surgery), comorbidities (hypertension, diabetes, congestive heart failure, previous myocardial infarction, peripheral vascular disease, atrial fibrillation, chronic obstructive pulmonary disease (COPD), chronic kidney disease (CKD), previous stroke, American Society of Anesthesiologists (ASA) score), prior medication (angiotensin-converting-enzyme inhibitors (ACEis) or angiotensin II receptor blockers (ARBs), beta-blockers, aspirin, statins, diuretics, use of contrast media within one week prior to surgery, nonsteroidal anti-inflammatory drugs (NSAIDs) except aspirin, vasopressors), surgical details (urgency of procedure, type of surgery, use and duration of cardiopulmonary bypass (CPB), use and duration of cross-clamp), intra- and postoperative fluids (fluid balance, total urinary output), intraoperative fluid details (use of cell saver, hypotensive episodes), intra- and postoperative medication (vasopressors, ACEi/ARBs, aminoglycosides, cyclosporine/tacrolimus, NSAIDs except aspirin, radiocontrast agents, vancomycin, diuretics), intraoperative complications (arrhythmias, bleeding, pulmonal complications, allergic reaction) and postoperative complications (hemodynamic instability, bleeding, re-operation, pneumonia, systemic inflammation).

**eTable 1: Patient perioperative and postoperative metrics**

|  | **All patients**  **(n = 3101)** | | **No CSA-AKI**  **(n = 2299)** | | | **CSA-AKI**  **(n = 802)** | | | **Significance** | | |
| --- | --- | --- | --- | --- | --- | --- | --- | --- | --- | --- | --- |
| **Perioperative metrics** | | | | | | | | | | | |
| ***Surgical details*** | | | | | | | | | | | |
| Urgency category | | | | | | | | | | | 0.439 |
| Elective | | 2978/3088 (96.4) | | | 2210/2288 (96.6) | | | 768/800 (96) | | |  |
| Emergency | | 110/3088 (3.6) | | | 78/2288 (3.4) | | | 32/800 (4) | | |  |
| CPB | | 3061/3101 (98.7) | | | 2266/2299 (98.6) | | | 795/802 (99.1) | | | 0.277 |
| CPB time, median (Q1, Q3), min (Missing n) | | 100 (75, 134)  (Missing 40) | | | 99 (75, 128)  (Missing 33) | | | 109 (77, 151)  (Missing 7) | | | <0.001 |
| Cross-Clamp | | 3055/3101 (98.5) | | | 2261/2299 (98.3) | | | 794/802 (99) | | | 0.235 |
| Cross-Clamp time, median (Q1, Q2), min (Missing n) | | 69 (51,94)  (Missing 46) | | | 68 (51, 92)  (Missing 38) | | | 72 (52, 104)  (Missing 8) | | | <0.001 |
| Duration of surgery, median (Q1, Q3), min (Missing n) | | 237 (190, 292)  (Missing 14) | | | 232 (189, 285)  (Missing 12) | | | 247 (198.8, 310)  (Missing 2) | | | <0.001 |
| **Fluids** | | | | | | | | | | | |
| Crystalloids, median (Q1, Q3), ml | | 1800 (1000, 2600) | | | 1860 (1000, 2700) | | | 1544 (1000, 2500) | | | 0.001 |
| Colloids, median (Q1, Q3), ml | | 0 (0, 500) | | | 0 (0, 500) | | | 0 (0, 100) | | | <0.001 |
| Erythrocytes, median (Q1, Q3), ml (Missing n) | | 0 (0, 300)  (Missing 115) | | | 0 (0, 280)  (Missing 102) | | | 0 (0, 470)  (Missing 13) | | | <0.001 |
| Thrombocytes, median (Q1, Q3), ml (Missing n) | | 0 (0, 0)  (Missing 149) | | | 0 (0, 0)  (Missing 131) | | | 0 (0, 0)  (Missing 18) | | | <0.001 |
| Fresh Frozen Plasma, median (Q1, Q3), ml (Missing n) | | 0 (0, 0)  (Missing 96) | | | 0 (0, 0)  (Missing 83) | | | 0 (0, 0)  (Missing 13) | | | 0.711 |
| Total blood loss, median (Q1, Q3), ml (Missing n) | | 300 (100, 500)  (Missing 292) | | | 350 (150, 500)  (Missing 212) | | | 200 (0, 500)  (Missing 80) | | | <0.001 |
| Total urinary output, median (Q1, Q3), ml (Missing n) | | 785 (400, 1300)  (Missing 85) | | | 850 (500, 1400)  (Missing 60) | | | 560 (300, 1080)  (Missing 25) | | | <0.001 |
| Transfusion (Yes/No) | | 1259/3027 (41.6) | | | 877/2235 (39.2) | | | 382/792 (48.2) | | | <0.001 |
| Transfusion, median (Q1, Q3), ml (Missing n) | | 0 (0, 529)  (Missing 167) | | | 0 (0, 500)  (Missing 146) | | | 0 (0, 610)  (Missing 21) | | | <0.001 |
| Fluid balance, median (Q1, Q3), ml (Missing n) | | 1024.5 (300, 1800)  (Missing 389) | | | 1000 (250, 1750)  (Missing 294) | | | 1100 (361.5, 1995) (Missing 95) | | | <0.001 |
| **Fluid details** | | | | | | | | | | | |
| Urinary catheter used | | 3060/3101 (98.7) | | | 2265/2299 (98.5) | | | 795/802 (99.1) | | | 0.280 |
| Cell saver used | | 1217/3101 (39.2) | | | 749/2299 (32.6) | | | 468/802 (58.4) | | | <0.001 |
| Episodes of hypotension | | 1033/3101 (33.3) | | | 708/2299 (30.8) | | | 325/802 (40.5) | | | <0.001 |
| **Use of vasopressors** | | | | | | | | | | | |
| Any | | 2314/3101 (74.6) | | | 1593/2299 (69.3) | | | 721/802 (89.9) | | | <0.001 |
| Norepinephrine | | 1713/3101 (55.2) | | | 1593/2299 (69.3) | | | 721/802 (89.9) | | | <0.001 |
| Epinephrine | | 406/3101 (13.1) | | | 245/2299 (10.7) | | | 161/802 (20.1) | | | <0.001 |
| Dobutamine | | 661/3101 (21.3) | | | 447/2299 (19.4) | | | 214/802 (26.7) | | | <0.001 |
| Vasopressin | | 157/3101 (5.1) | | | 90/2299 (3.9) | | | 67/802 (8.4) | | | <0.001 |
| Other | | 668/3101 (21.5) | | | 451/2299 (19.69) | | | 217/802 (27.1) | | | <0.001 |
| **Use of nephrotoxic agents** | | | | | | | | | | | |
| Any | | 465/3101 (15) | | | 302/2299 (13.1) | | | 163/802 (20.3) | | | <0.001 |
| ACEi/ARBs | | 11/3101 (0.4) | | | 9/2299 (0.4) | | | 2/802 (0.2) | | | 0.739 |
| Aminoglycosides | | 105/3101 (3.4) | | | 72/2299 (3.1) | | | 33/802 (4.1) | | | 0.212 |
| Amphotericin B | | 0/3101 (0) | | | 0/2299 (0) | | | 0/802 (0) | | | 1.000 |
| Cyclosporine/Tacrolimus | | 0/3101 (0) | | | 0/2299 (0) | | | 0/802 (0) | | | 1.000 |
| NSAIDs (except Aspirin/ASS) | | 79/3101 (2.5) | | | 61/2299 (2.7) | | | 18/802 (2.2) | | | 0.603 |
| Radiocontrast agents | | 17/3101 (0.5) | | | 9(2299 (0.4) | | | 8/802 (1) | | | 0.054 |
| Vancomycin | | 234/3101 (7.5) | | | 132/2299 (5.7) | | | 102/802 (12.7) | | | <0.001 |
| Other | | 123/3101 (4) | | | 76/2299 (3.3) | | | 47/802 (5.9) | | | 0.002 |
| **Use of diuretics** | | | | | | | | | | | |
| Any | | 904/3101 (29.2) | | | 684/2299 (29.8) | | | 220/802 (27.4) | | | 0.223 |
| **Intraoperative complications** | | | | | | | | | | | |
| Any | | 573/3101 (18.5) | | | 362/2299 (15.7) | | | 211/802 (26.3) | | | <0.001 |
| Cardiopulmonary resuscitation | | 15/3101 (0.5) | | | 9/2299 (0.4) | | | 6/802 (0.7) | | | 0.237 |
| Arrhythmias | | 223/3101 (7.2) | | | 147/2299 (6.4) | | | 76/802 (9.5) | | | 0.005 |
| Bleeding | | 390/3101 (12.6) | | | 237/2299 (10.3) | | | 153/802 (19.1) | | | <0.001 |
| Pulmonary complications | | 14/3101 (0.5) | | | 4/2299 (0.2) | | | 10/802 (1.2) | | | <0.001 |
| Allergic reaction | | 6/3101 (0.2) | | | 3/2299 (0.1) | | | 3/802 (0.4) | | | 0.183 |
| Other | | 58/3101 (1.9) | | | 38/2299 (1.7) | | | 20/802 (2.5) | | | 0.132 |
| **Postoperative metrics** | | | | | | | | | | | |
| Apache II, median (Q1, Q3) | | 10 (7, 15) | | 9 (6, 13) | | | 13 (9, 18) | | | <0.001 | |
| SAPS II, median (Q1, Q3) | | 21 (13, 30) | | 18 (12, 26) | | | 28 (20, 39) | | | <0.001 | |
| **Fluids (cumulative at day 3)** | | | | | | | | | | | |
| Crystalloids, median (Q1, Q3), ml (Missing n) | | 6000 (4000, 8450)  (Missing 25) | | 5856 (3963, 8335) (Missing 22) | | | 6245 (4188.5, 8853) (Missing 3) | | | 0.001 | |
| Colloids, median (Q1, Q3), ml | | 0 (0, 500) | | 0 (0, 500) | | | 50 (0, 500) | | | <0.001 | |
| Erythrocytes, median (Q1, Q3), ml (Missing n) | | 0 (0, 300)  (Missing 113) | | 0 (0, 300)  (Missing 101) | | | 0 (0, 511.2)  (Missing 12) | | | <0.001 | |
| Thrombocytes, median (Q1, Q3), ml (Missing n) | | 0 (0, 0)  (Missing 174) | | 0 (0, 0)  (Missing 153) | | | 0 (0, 0)  (Missing 21) | | | <0.001 | |
| Fresh Frozen Plasma, median (Q1, Q3), ml (Missing n) | | 0 (0, 0)  (Missing 144) | | 0 (0. 0)  (Missing 128) | | | 0 (0, 0)  (Missing 16) | | | 0.913 | |
| Total blood loss, median (Q1, Q3), ml (Missing n) | | 510 (300, 900)  (Missing 42) | | 480 (260, 850)  (Missing 36) | | | 700 (400, 1190.2)  (Missing 6) | | | <0.001 | |
| Total urinary output, median (Q1, Q3), ml (Missing n) | | 5300 (3572.5, 7050) (Missing 23) | | 5544 (3952.5, 7200) (Missing 20) | | | 4435 (2845, 6600)  (Missing 3) | | | <0.001 | |
| Transfusion (Yes/No) | | 1316/2998 (43.9) | | 918/2206 (41.6) | | | 398/792 (50.3) | | | <0.001 | |
| Transfusion, median (Q1, Q3), ml (Missing n) | | 0 (0, 560) (Missing 178) | | 0 (0, 500)  (Missing 156) | | | 0 (0, 700)  (Missing 22) | | | <0.001 | |
| Fluid balance, median (Q1, Q3), ml (Missing n) | | 865 (-712, 2400)  (Missing 190) | | 665 (-900, 2108.5) (Missing 167) | | | 1590 (-127.5, 3565) (Missing 23) | | | <0.001 | |
| **Use of vasopressors** | | | | | | | | | | | |
| Any | | 1975/3101 (63.7) | | 1339/2299 (58.2) | | | 636/802 (79.3) | | | <0.001 | |
| Norepinephrine | | 1515/3101 (48.9) | | 989/2299 (43) | | | 526/802 (65.6) | | | <0.001 | |
| Epinephrine | | 345/3101 (11.1) | | 206/2299 (9) | | | 139/802 (17.3) | | | <0.001 | |
| Dobutamine | | 573/3101 (18.5) | | 347/2299 (15.1) | | | 226/802 (28.2) | | | <0.001 | |
| Vasopressin | | 64/3101 (2.1) | | 27/2299 (1.2) | | | 37/802 (4.6) | | | <0.001 | |
| Other | | 339/3101 (10.9) | | 242/2299 (10.5) | | | 97/802 (12.1) | | | 0.237 | |
| **Use of nephrotoxic agents** | | | | | | | | | | | |
| Any | | 1182/3093 (38.2) | | 824/2292 (36) | | | 358/801 (44.7) | | | <0.001 | |
| ACEi/ARBs | | 368/3100 (11.9) | | 227/2298 (9.9) | | | 141/802 (17.6) | | | <0.001 | |
| Aminoglycosides | | 26/3100 (0.8) | | 12/2298 (0.5) | | | 14/802 (1.7) | | | 0.002 | |
| Amphotericin B | | 0/3100 (0.8) | | 0/2298 (0) | | | 0/802 (0) | | | 1.000 | |
| Cyclosporine/Tacrolimus | | 5/3100 (0.2) | | 4/2298 (0.2) | | | 1/802 (0.1) | | | 1.000 | |
| NSAIDs (except Aspirin/ASS) | | 666/3100 (21.5) | | 519/2298 (22.6) | | | 147/802 (18.3) | | | 0.012 | |
| Radiocontrast agents | | 48/3100 (1.5) | | 21/2298 (0.9) | | | 27/802 (3.4) | | | <0.001 | |
| Vancomycin | | 235/3100 (7.6) | | 131/2298 (5.7) | | | 104/802 (13) | | | <0.001 | |
| Other | | 125/3100 (4) | | 63/2298 (2.7) | | | 62/802 (7.7) | | | <0.001 | |
| **Use of diuretics** | | | | | | | | | | | |
| Any | | 1939/3091 (62.7) | | 1331/2291 (58.1) | | | 608/800 (76) | | | <0.001 | |
| Immediately postop. | | 556/3100 (17.9) | | 376/2298 (16.4) | | | 180/802 (22.4) | | | <0.001 | |
| Day 1 | | 1156/3100 (37.3) | | 811/2298 (35.3) | | | 345/802 (43) | | | <0.001 | |
| Day 2 | | 1253/3100 (40.4) | | 833/2298 (36.2) | | | 420/802 (52.4) | | | <0.001 | |
| Day 3 | | 1168/3100 (37.7) | | 764/2298 (33.2) | | | 404/802 (50.4) | | | <0.001 | |
| **Postoperative complications** | | | | | | | | | | | |
| Hemodynamic instability | | 461/3100 (14.9) | | 226/2298 (9.8) | | | 235/802 (29.3) | | | <0.001 | |
| Bleeding | | 589/3100 (19) | | 355/2298 (15.4) | | | 234/802 (29.2) | | | <0.001 | |
| Re-operation | | 112/3100 (3.6) | | 46/2298 (2) | | | 66/802 (8.2) | | | <0.001 | |
| Pneumonia | | 60/3100 (1.9) | | 26/2298 (1.1) | | | 34/802 (4.2) | | | <0.001 | |
| Systemic inflammation | | 36/3100 (1.2) | | 4/2298 (0.2) | | | 32/802 (4) | | | <0.001 | |
| Other | | 201/3100 (6.5) | | 111/2298 (4.8) | | | 90/802 (11.2) | | | <0.001 | |
| **Postoperative RRT** | | | | | | | | | | | |
| Any indication | | 101/3097 (3.3) | | 1/2295 (0) | | | 100/802 (12.5) | | | <0.001 | |
| Pulmonary edema | | 18/3100 (0.6) | | 0/2298 (0) | | | 18/802 (2.2) | | | <0.001 | |
| Hypervolemia | | 29/3100 (0.9) | | 0/2298 (0) | | | 29/802 (3.6) | | | <0.001 | |
| Electrolyte derangement | | 50/3100 (1.6) | | 0/2298 (0) | | | 50/802 (6.2) | | | <0.001 | |
| Uremia | | 32/3100 (1) | | 0/2298 (0) | | | 32/802 (4) | | | <0.001 | |
| Anuria | | 67/3100 (2.2) | | 1/2298 (0) | | | 66/802 (4) | | | <0.001 | |
| Others | | 31/3100 (1) | | 0/2298 (0) | | | 31/802 (3.9) | | | <0.001 | |

**Abbreviations:** CSA-AKI, cardiac surgery associated – acute kidney injury; CPB, cardiac pulmonal bypass, ACEi/ARB, angiotensin-converting-enzyme inhibitor / aldosterone receptor blocker; NSAIDs, non-steroidal ant-inflammatory drugs, Apache, acute physiology and chronic heart evaluation; SAPS, simplified acute physiology score

**eTable 2**: KDIGO stage reached, all patients divided by AKI y/n, AKI stage, transient vs persistent AKI

| **KDIGO Stage** | **CSA-AKI duration** | **Count** |
| --- | --- | --- |
| No AKI | - | 2274 |
| KDIGO 1 | Transient (< 48h) | 387 |
| KDIGO 1 | Persistent (> 48h) | 100 |
| KDIGO 2 | Transient (< 48h) | 131 |
| KDIGO 2 | Persistent (> 48h) | 80 |
| KDIGO 3 | Transient (< 48h) | 5 |
| KDIGO 3 | Persistent (> 48h) | 99 |

**Abbreviations:** CSA-AKI, cardiac surgery associated – acute kidney injury; KDIGO, Kidney Disease Improving Global Outcomes

**eTable 3: Multivariable regression analysis for CSA-AKI development**

| **Variable** | **OR (95% CI)** | **Significance** |
| --- | --- | --- |
| ***Baseline characteristics*** | | |
| **Demographics** | | |
| Age (year) | 1.026 ( 1.016 , 1.037 ) | <0.001 |
| BMI (kg/m^2^) | 1.005 ( 0.996 , 1.013 ) | 0.2889 |
| Serum-creatinine (mg/dl) | 3.406 ( 2.399 , 4.837 ) | <0.001 |
| UN geoscheme |  |  |
| Africa vs Europe | 1.761 ( 0.347 , 8.934 ) | 0.4948 |
| Asia vs Europe | 1.554 ( 0.312 , 7.724 ) | 0.5902 |
| North America vs Europe | 2.115 ( 0.205 , 21.796 ) | 0.5290 |
| South America vs Europe | 1.394 ( 0.123 , 15.838 ) | 0.7887 |
| Health expenditure group |  |  |
| Health expenditure (medium vs low) | 1.089 ( 0.245 , 4.846 ) | 0.9110 |
| Health expenditure (high vs low) | 2.401 ( 0.392 , 14.715 ) | 0.3435 |
| **Comorbidities** | | |
| Hypertension (yes vs. no) | 1.484 ( 1.142 , 1.928 ) | 0.0031 |
| Diabetes (yes vs. no) | 1.348 ( 1.079 , 1.685 ) | 0.0086 |
| Atrial fibrillation/flutter (yes vs. no) | 1.525 ( 1.161 , 2.003 ) | 0.0086 |
| ASA score |  |  |
| ASA score (2 vs 1) | 1.049 ( 0.344 , 3.2 ) | 0.9324 |
| ASA score (3 vs 1) | 1 ( 0.33 , 3.034 ) | 0.9999 |
| ASA score (4 vs 1) | 1.225 ( 0.387 , 3.881 ) | 0.7298 |
| **Medication** | | |
| Diuretics (yes vs. no) | 1.186 ( 0.953 , 1.476 ) | 0.1269 |
| Use of contrast media 1w pr. Surg. (yes vs no) | 1.201 ( 0.923 , 1.561 ) | 0.1721 |
| NSAIDs (except Aspirin) | 0.785 ( 0.478 , 1.29 ) | 0.3390 |
| ***Perioperative characteristics*** | | |
| **Surgical details** | | |
| CPB time (min) | 1.005 ( 1.003 , 1.007 ) | <0.001 |
| **Fluids** | | |
| Urinary output (intra (dl) | 0.972 ( 0.955 , 0.99 ) | <0.001 |
| **Fluid details** | | |
| Cell saver used (yes vs. no) | 1.635 ( 1.188 , 2.251 ) | 0.0026 |
| **Use of vasopressors** | | |
| Vasopressors (intra) (yes vs. no) | 1.225 ( 0.872 , 1.721 ) | 0.2411 |
| **Use of nephrotoxic agents** | | |
| Aminoglycosides (intra) (yes vs. no) | 1.465 ( 0.612 , 3.503 ) | 0.3908 |
| **Use of diuretics** | | |
| Diuretics (intra) (yes vs. no) | 1.67 ( 1.238 , 2.254 ) | 0.0008 |
| **Intraoperative complications** | | |
| Bleeding (intra) (yes vs. no) | 1.628 ( 1.16 , 2.284 ) | 0.0048 |
| Pulmonal complications (intra) (yes vs. no) | 5.562 ( 1.134 , 27.283 ) | 0.0345 |
| Allergic reaction | 2.444 ( 0.246 , 24.241 ) | 0.4451 |
| ***Postoperative characteristics*** | | |
| **Fluids (cumulative to. day 3)** | | |
| Fluid balance (post) (l) | 1.144 ( 1.09 , 1.201 ) | <.0001 |
| Urinary output (post) (dl) | 0.999 ( 0.994 , 1.004 ) | 0.7184 |
| **Use of nephrotoxic agents** | | |
| NSAIDs (exc. Aspirin/ASS) (post) (yes vs. no) | 1.312 ( 0.933 , 1.843 ) | 0.1184 |
| **Use of diuretics** | | |
| Diuretics (post) (yes vs. no) | 2.067 ( 1.579 , 2.705 ) | <.0001 |
| **Postoperative complications** | | |
| Hemodynamic instability (post) (yes vs. no) | 1.648 ( 1.225 , 2.217 ) | 0.0010 |
| Bleeding (post) (yes vs. no) | 1.62 ( 1.21 , 2.168 ) | 0.0012 |
| Re-operation (yes vs. no) | 1.973 ( 1.175 , 3.311 ) | 0.0101 |
| Pneumonia (post) (yes vs. no) | 3.08 ( 1.453 , 6.532 ) | 0.0034 |
| Systemic inflammation (post) (yes vs. no) | 2.366 ( 0.573 , 9.761 ) | 0.2337 |

**Abbreviations:** CSA-AKI, cardiac surgery associated – acute kidney injury; NSAIDs, non-steroidal ant-inflammatory drugs; ASA, American Society of Anesthesiologists; CPB, cardio pulmonal bypass

**eTable 4: Multivariable regression analysis for development of moderate/severe CSA-AKI (KDIGO 2/3)**

| **Variable** | **OR (95% CI)** | **Significance** |
| --- | --- | --- |
| ***Baseline characteristics*** | | |
| **Demographics** | | |
| Age (year) | 1.003 ( 0.985 , 1.021 ) | 0.7408 |
| BMI (kg/m^2^) | 1.009 ( 0.994 , 1.024 ) | 0.2298 |
| Serum-creatinine (mg/dl) | 1.488 ( 0.974 , 2.273 ) | 0.0658 |
| Health expenditure group |  |  |
| Health expenditure (medium vs low) | 3.478 ( 1.077 , 11.226 ) | 0.0163 |
| Health expenditure (high vs low) | 3.588 ( 1.266 , 10.165 ) | 0.0371 |
| **Comorbidities** | | |
| Diabetes (yes vs. no) | 1.6 ( 1.088 , 2.351 ) | 0.0169 |
| Atrial fibrillation/flutter (yes vs. no) | 1.075 ( 0.702 , 1.647 ) | 0.7394 |
| COPD (yes vs. no) | 1.361 ( 0.761 , 2.434 ) | 0.2981 |
| ASA score |  |  |
| ASA score (2 vs 1) | 0.751 ( 0.109 , 5.17 ) | 0.7704 |
| ASA score (3 vs 1) | 0.819 ( 0.122 , 5.483 ) | 0.8368 |
| ASA score (4 vs 1) | 1.406 ( 0.202 , 9.803 ) | 0.7305 |
| **Medication** | | |
| Statins (yes vs. no) | 0.783 ( 0.527 , 1.162 ) | 0.2240 |
| Use of contrast media 1w pr. surg. (yes vs. no) | 1.106 ( 0.71 , 1.725 ) | 0.6548 |
| ***Perioperative characteristics*** | | |
| **Surgical details** | | |
| **Fluids** | | |
| Urinary output (intra (dl) | 1.016 ( 0.986 , 1.047 ) | 0.3021 |
| **Fluid details** | | |
| Cell saver used (yes vs. no) | 1.094 ( 0.658 , 1.82 ) | 0.7276 |
| **Use of nephrotoxic agents** | | |
| Aminoglycosides (intra) (yes vs. no) | 1.739 ( 0.439 , 6.884 ) | 0.4299 |
| Vancomycin (intra) (yes vs. no) | 1.251 ( 0.418 , 3.748 ) | 0.6889 |
| **Intraoperative complications** | | |
| Bleeding (intra) (yes vs. no) | 2.53 ( 1.499 , 4.271 ) | 0.0005 |
| Pulmonary complications (intra) (yes vs. no) | 24.832 ( 1.991 , 309.667 ) | 0.0127 |
| ***Postoperative characteristics*** | | |
| **Fluids (cumulative to day 3)** | | |
| Fluid balance (post) (l) | 1.146 ( 1.06 , 1.239 ) | 0.0007 |
| Urinary output (post) (dl) | 0.994 ( 0.986 , 1.002 ) | 0.1230 |
| ***Use of vasopressors*** | | |
| Vasopressors (post) (yes vs. no) | 0.978 ( 0.568 , 1.685 ) | 0.9361 |
| **Postoperative complications** | | |
| Hemodynamic instability (post) (yes vs. no) | 1.707 ( 1.078 , 2.704 ) | 0.0226 |
| Re-operation (yes vs. no) | 2.371 ( 1.205 , 4.665 ) | 0.0125 |
| Systemic inflammation (post) (yes vs. no) | 2.344 ( 0.733 , 7.497 ) | 0.1506 |

**Abbreviations:** CSA-AKI, cardiac surgery associated – acute kidney injury; BMI, body mass index; COPD, chronic obstructive pulmonal disease; ASA, American Society of Anesthesiologists; CPB, cardio pulmonal bypass

**eTable 5: Multivariable regression analysis for persistent CSA-AKI**

| **Variable** | **OR (95% CI)** | **Significance** |
| --- | --- | --- |
| ***Baseline characteristics*** | | |
| **Demographics** | | |
| Age (year) | 1.021 ( 1.001 , 1.041 ) | 0.0374 |
| BMI (kg/m^2^) | 1.008 ( 0.998 , 1.018 ) | 0.1027 |
| Serum-creatinine (mg/dl) | 1.94 ( 1.182 , 3.187 ) | 0.0089 |
| UN geoscheme |  |  |
| Africa vs Europe | 3.121 ( 0.641 , 15.191 ) | 0.1584 |
| Asia vs Europe | 1.095 ( 0.476 , 2.521 ) | 0.8309 |
| North America vs Europe | 3.562 ( 0.59 , 21.488 ) | 0.1657 |
| South America vs Europe | 0.242 ( 0.016 , 3.673 ) | 0.3062 |
| **Comorbidities** | | |
| Hypertension (yes vs. no) | 1.487 ( 0.883 , 2.504 ) | 0.1351 |
| Congestive heart failure (yes vs no) | 0.745 ( 0.458 , 1.209 ) | 0.2329 |
| CKD baseline (yes vs. no) | 2.58 ( 1.495 , 4.452 ) | 0.0007 |
| Previous Stroke (yes vs. no) | 0.616 ( 0.302 , 1.257 ) | 0.1828 |
| ASA score |  |  |
| ASA score (2 vs 1) | 0.488 ( 0.061 , 3.9 ) | 0.4984 |
| ASA score (3 vs 1) | 0.626 ( 0.08 , 4.887 ) | 0.6544 |
| ASA score (4 vs 1) | 1.152 ( 0.142 , 9.318 ) | 0.8945 |
| **Medication** | | |
| Aspirin (yes vs. no) | 0.681 ( 0.456 , 1.017 ) | 0.0601 |
| NSAIDs (except Aspirin) (yes vs. no) | 0.515 ( 0.194 , 1.365 ) | 0.1817 |
| Diuretics | 0.999 ( 0.672 , 1.485 ) | 0.9964 |
| ***Perioperative characteristics*** | | |
| **Surgical details** | | |
| CPB time (min) | 1.008 ( 1.004 , 1.012 ) | <0.001 |
| **Fluids** | | |
| Urinary output (intra (dl) | 0.982 ( 0.95 , 1.014 ) | 0.2715 |
| **Fluid details** | | |
| **Use of vasopressors** | | |
| Vasopressors (intra) (yes vs. no) | 1.57 ( 0.767 , 3.212 ) | 0.2165 |
| **Use of nephrotoxic agents** | | |
| Aminoglycosides (intra) (yes vs. no) | 5.055 ( 1.207 , 21.163 ) | 0.0266 |
| **Use of diuretics** | | |
| Diuretics (intra) (yes vs. no) | 1.384 ( 0.834 , 2.295 ) | 0.2080 |
| **Intraoperative complications** | | |
| Bleeding (intra) (yes vs. no) | 1.096 ( 0.624 , 1.923 ) | 0.7499 |
| Pulmonal complications (intra) (yes vs. no) | 2.776 ( 0.507 , 15.198 ) | 0.2388 |
| ***Postoperative characteristics*** | | |
| **Fluids (cumulative to day 3)** | | |
| Fluid balance (post) (l) | 1.126 ( 1.038 , 1.222 ) | 0.0042 |
| Urinary output (post) (dl) | 0.995 ( 0.987 , 1.003 ) | 0.2634 |
| **Use of nephrotoxic agents** | | |
| Aminoglycosides (post) (yes vs. no) | 1.543 ( 0.278 , 8.55 ) | 0.6194 |
| **Use of diuretics** | | |
| Diuretics (post) (yes vs. no) | 1.581 ( 0.918 , 2.722 ) | 0.0982 |
| **Postoperative complications** | | |
| Hemodynamic instability (post) (yes vs. no) | 1.357 ( 0.865 , 2.128 ) | 0.1840 |
| Bleeding (post) (yes vs. no) | 1.238 ( 0.772 , 1.986 ) | 0.3757 |
| Re-operation (yes vs. no) | 3.357 ( 1.676 , 6.725 ) | 0.0007 |
| Systemic inflammation (post) (yes vs. no) | 4.271 ( 1.308 , 13.95 ) | 0.0163 |

**Abbreviations:** CSA-AKI, cardiac surgery associated – acute kidney injury; BMI, body mass index; ASA, American Society of Anesthesiologists, CPB, cardio pulmonal bypass; NSAIDs, non-steroidal ant-inflammatory drugs

**eTable 6: ICU death Generalized Mixed linear Model logit as Link function Center as random effect**

| **Variable** | **OR (95% CI)** | **Significance** |
| --- | --- | --- |
| Age, y | 1.031 ( 0.995 , 1.069 ) | 0.0883 |
| BMI | 1.008 ( 0.999 , 1.017 ) | 0.0970 |
| Serum-creatinine | 0.695 ( 0.314 , 1.54 ) | 0.3706 |
| Hypertension  (Yes vs. No) | 0.739 ( 0.329 , 1.659 ) | 0.4637 |
| Diabetes  (Yes vs. No) | 0.384 ( 0.176 , 0.842 ) | 0.0168 |
| Previous myocardial infarction  (Yes vs. No) | 0.927 ( 0.433 , 1.985 ) | 0.8453 |
| COPD  (Yes vs. No) | 3.609 ( 1.622 , 8.031 ) | 0.0017 |
| CKD (GFR <60 ml/min)  Yes vs. No | 2.152 ( 0.827 , 5.602 ) | 0.1165 |
| PO-AKI stages |  | <.0001 |
| KDIGO 2/3 vs. KDIGO 1 | 25.488 ( 8.259 , 78.664 ) | <.0001 |
| No AKI vs. KDIGO 1 | 0.575 ( 0.172 , 1.928 ) | 0.3702 |

**eTable 7: Hospital death Generalized Mixed linear Model logit as Link function Center as random effect**

| **Variable** | **OR (95% CI)** | **Significance** |
| --- | --- | --- |
| Age, y | 1.038 ( 1.008 , 1.068 ) | 0.0135 |
| BMI | 1.007 ( 0.998 , 1.016 ) | 0.1165 |
| Serum-creatinine | 0.871 ( 0.492 , 1.54 ) | 0.6342 |
| Hypertension  (Yes vs. No) | 0.972 ( 0.484 , 1.95 ) | 0.9354 |
| Diabetes  (Yes vs. No) | 0.401 ( 0.212 , 0.76 ) | 0.0051 |
| Previous myocardial infarction  (Yes vs. No) | 1.055 ( 0.574 , 1.939 ) | 0.8634 |
| COPD  (Yes vs. No) | 2.607 ( 1.327 , 5.125 ) | 0.0055 |
| CKD (GFR <60 ml/min)  Yes vs. No | 1.419 ( 0.646 , 3.117 ) | 0.3834 |
| PO-AKI stages |  | <.0001 |
| KDIGO 2/3 vs. KDIGO 1 | 14.07 ( 6.061 , 32.66 ) | <.0001 |
| No AKI vs. KDIGO 1 | 0.499 ( 0.206 , 1.208 ) | 0.1235 |

**eTable 8: Death until day 90 Generalized Mixed linear Model logit as Link function Center as random effect**

| **Variable** | **OR (95% CI)** | **Significance** |
| --- | --- | --- |
| Age, y | 1.047 ( 1.019 , 1.075 ) | 0.0007 |
| BMI | 1.003 ( 0.995 , 1.012 ) | 0.4489 |
| Serum-creatinine | 0.789 ( 0.449 , 1.387 ) | 0.4106 |
| Hypertension  (Yes vs. No) | 0.955 ( 0.517 , 1.765 ) | 0.8840 |
| Diabetes  (Yes vs. No) | 0.657 ( 0.392 , 1.1 ) | 0.1103 |
| Previous myocardial infarction  (Yes vs. No) | 0.875 ( 0.512 , 1.496 ) | 0.6254 |
| COPD  (Yes vs. No) | 2.406 ( 1.35 , 4.289 ) | 0.0029 |
| CKD (GFR <60 ml/min)  Yes vs. No | 1.819 ( 0.94 , 3.521 ) | 0.0756 |
| PO-AKI stages |  | <.0001 |
| KDIGO 2/3 vs. KDIGO 1 | 6.831 ( 3.55 , 13.148 ) | <.0001 |
| No AKI vs. KDIGO 1 | 0.381 ( 0.194 , 0.748 ) | 0.0051 |

**eTable 9: RRT in Hospital Generalized Mixed linear Model logit as Link function Center as random effect**

| **Variable** | **OR (95% CI)** | **Significance** |
| --- | --- | --- |
| Age, y | 0.989 ( 0.963 , 1.017 ) | 0.4489 |
| BMI | 0.996 ( 0.983 , 1.01 ) | 0.5869 |
| Serum-creatinine | 2.232 ( 1.325 , 3.76 ) | 0.0026 |
| Hypertension  (Yes vs. No) | 0.992 ( 0.458 , 2.145 ) | 0.9831 |
| Diabetes  (Yes vs. No) | 0.751 ( 0.403 , 1.4 ) | 0.3681 |
| Previous myocardial infarction  (Yes vs. No) | 0.676 ( 0.342 , 1.337 ) | 0.2604 |
| COPD  (Yes vs. No) | 2.44 ( 1.152 , 5.164 ) | 0.0198 |
| CKD (GFR <60 ml/min)  Yes vs. No | 1.908 ( 0.91 , 3.997 ) | 0.0870 |
| PO-AKI stages |  | <.0001 |
| KDIGO 2/3 vs. KDIGO 1 | 19.962 ( 8.53 , 46.719 ) | <.0001 |
| No AKI vs. KDIGO 1 | 0.091 ( 0.023 , 0.36 ) | 0.0006 |

**eTable 10: ICU stay Mixed linear Model Center as random effect**

| **Variable** | **Estimate (95% CI)** | **Significance** |
| --- | --- | --- |
| Age, y | -0.001 ( -0.025 , 0.023 ) | 0.0058 |
| BMI | -0.007 ( -0.028 , 0.014 ) | 0.9242 |
| Serum-creatinine | 1.302 ( 0.494 , 2.11 ) | 0.5165 |
| Hypertension  (Yes vs. No) | -0.373 ( -1.013 , 0.267 ) | 0.0016 |
| Diabetes  (Yes vs. No) | 0.315 ( -0.26 , 0.89 ) | 0.2833 |
| Previous myocardial infarction  (Yes vs. No) | -0.135 ( -0.711 , 0.441 ) | 0.6455 |
| COPD  (Yes vs. No) | 0.408 ( -0.523 , 1.339 ) | 0.3904 |
| CKD (GFR <60 ml/min)  Yes vs. No | -0.086 ( -1.01 , 0.838 ) | 0.8553 |
| PO-AKI stages |  | <.0001 |
| KDIGO 2/3 vs. KDIGO 1 | 3.187 ( 2.063 , 4.311 ) | <.0001 |
| No AKI vs. KDIGO 1 | -0.915 ( -1.648 , -0.181 ) | 0.0146 |

**eTable 11: Hospital stay Mixed linear Model Center as random effect**

| **Variable** | **Estimate (95% CI)** | **Significance** |
| --- | --- | --- |
| Age, y | 14.234 ( 7.986 , 20.482 ) | 0.8687 |
| BMI | 0.006 ( -0.063 , 0.074 ) | 0.0758 |
| Serum-creatinine | 0.054 ( -0.006 , 0.113 ) | 0.0132 |
| Hypertension  (Yes vs. No) | 2.861 ( 0.6 , 5.122 ) | 0.5470 |
| Diabetes  (Yes vs. No) | 1.647 ( 0.028 , 3.265 ) | 0.0461 |
| Previous myocardial infarction  (Yes vs. No) | 1.071 ( -0.549 , 2.692 ) | 0.1950 |
| COPD  (Yes vs. No) | 0.806 ( -1.817 , 3.428 ) | 0.5469 |
| CKD (GFR <60 ml/min)  Yes vs. No | 1.669 ( -0.932 , 4.269 ) | 0.2085 |
| PO-AKI stages |  | <.0001 |
| KDIGO 2/3 vs. KDIGO 1 | 4.974 ( 1.833 , 8.116 ) | 0.0019 |
| No AKI vs. KDIGO 1 | -2.051 ( -4.119 , 0.017 ) | 0.0519 |

**eFigure 1**: RRT in ICU, all patients divided by AKI stage and transient vs persistent AKI

**
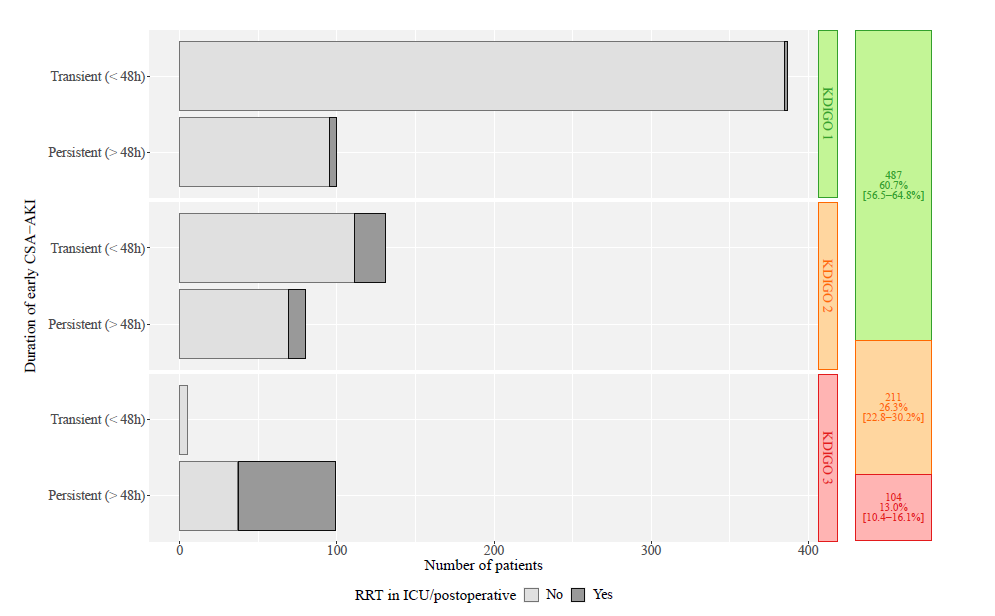
**

Statistical representation of within ICU RRT use of CSA-AKI patients separated by the highest reached stages within 72h postoperative, as well as AKI-duration. The X-axis on the right side represents the number of patients and percentage in each AKI stage, identified by colour (green as stage 1, orange as stage 2 and red as stage 30). (CSA-AKI=cardiac surgery associated acute kidney injury // KDIGO=kidney disease improving global outcomes)

**eFigure 2**: RRT in hospital, all patients divided by AKI stage and transient vs persistent AKI

**
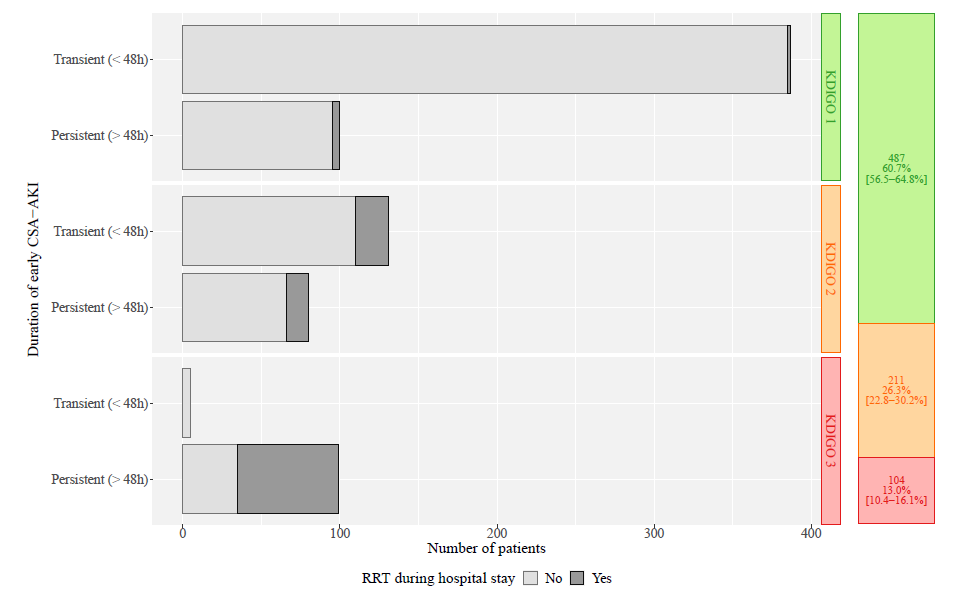
**

Statistical representation of within hospitalization RRT use of CSA-AKI patients separated by the highest reached stages within 72h postoperative, as well as AKI-duration. The X-axis on the right side represents the number of patients and percentage in each AKI stage, identified by colour (green as stage 1, orange as stage 2 and red as stage 30). (CSA-AKI=cardiac surgery associated acute kidney injury // KDIGO=kidney disease improving global outcomes).

**eFigure 3**: ICU mortality, all patients divided by AKI stage and transient vs persistent AKI

**
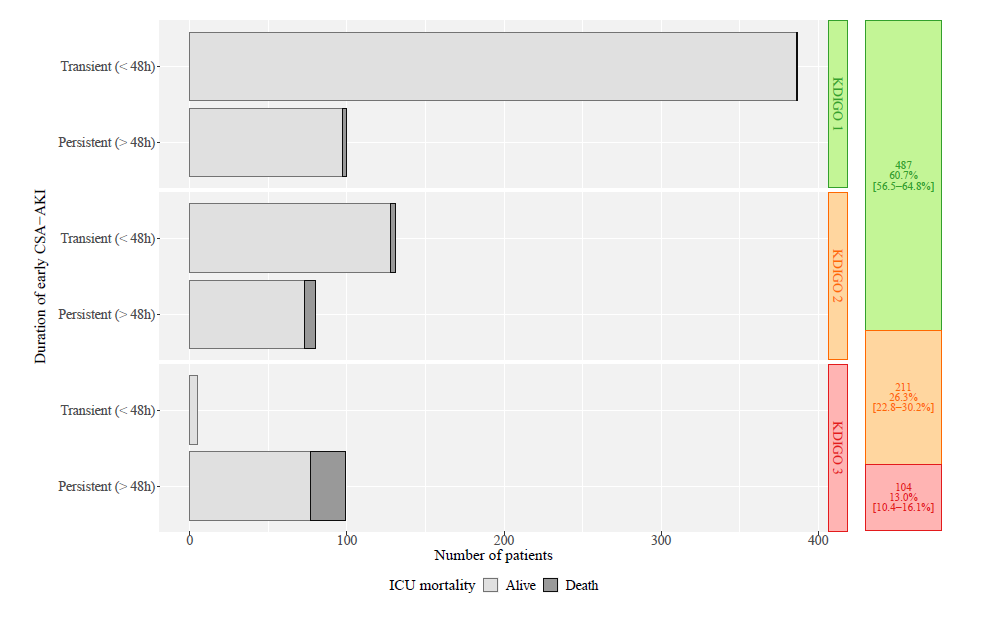
**

Statistical representation of within ICU mortality of CSA-AKI patients separated by the highest reached stages within 72h postoperative, as well as AKI-duration. The X-axis on the right side represents the number of patients and percentage in each AKI stage, identified by colour (green as stage 1, orange as stage 2 and red as stage 30). (CSA-AKI=cardiac surgery associated acute kidney injury // KDIGO=kidney disease improving global outcomes)
